# High Density Mapping of Quantitative Trait Loci Conferring Gluten Strength in Canadian Durum Wheat

**DOI:** 10.3389/fpls.2020.00170

**Published:** 2020-03-04

**Authors:** Yuefeng Ruan, Bianyun Yu, Ron E. Knox, Asheesh K. Singh, Ron DePauw, Richard Cuthbert, Wentao Zhang, Isabelle Piche, Peng Gao, Andrew Sharpe, Pierre Fobert

**Affiliations:** ^1^ Swift Current Research and Development Centre, Agriculture and Agri-Food Canada, Swift Current, SK, Canada; ^2^ Aquatic and Crop Resource Development, National Research Council Canada, Saskatoon, SK, Canada; ^3^ Aquatic and Crop Resource Development, National Research Council Canada, Ottawa, ON, Canada

**Keywords:** durum wheat, gluten strength, sodium dodecyl sulphate-sedimentation volume, quantitative trait loci, single nucleotide polymorphism

## Abstract

Gluten strength is one of the factors that determine the end-use quality of durum wheat and is an important breeding target for this crop. To characterize the quantitative trait loci (QTL) controlling gluten strength in Canadian durum wheat cultivars, a population of 162 doubled haploid (DH) lines segregating for gluten strength and derived from cv. Pelissier × cv. Strongfield was used in this study. The DH lines, parents, and controls were grown in 3 years and two seeding dates in each year and gluten strength of grain samples was measured by sodium dodecyl sulfate (SDS)-sedimentation volume (SV). With a genetic map created by genotyping the DH lines using the Illumina Infinium iSelect Wheat 90K SNP (single nucleotide polymorphism) chip, QTL contributing to gluten strength were detected on chromosome 1A, 1B, 2B, and 3A. Two major and stable QTL detected on chromosome 1A (*QGlu.spa-1A*) and 1B (*QGlu.spa-1B.1*) explaining 13.7–18.7% and 25.4–40.1% of the gluten strength variability respectively were consistently detected over 3 years, with the trait increasing alleles derived from Strongfield. Putative candidate genes underlying the major QTL were identified. Two novel minor QTL (*QGlu.spa-3A.1* and *QGlu.spa-3A.2*) with the trait increasing allele derived from Pelissier were mapped on chromosome 3A explaining up to 8.9% of the phenotypic variance; another three minor QTL (*QGlu.spa-2B.1*, *QGlu.spa-2B.2*, and *QGlu.spa-2B.3*) located on chromosome 2B explained up to 8.7% of the phenotypic variance with the trait increasing allele derived from Pelissier. *QGlu.spa-2B.1* is a new QTL and has not been reported in the literature. Multi-environment analysis revealed genetic (QTL) × environment interaction due to the difference of effect in magnitude rather than the direction of the QTL. Eleven pairs of digenic epistatic QTL were identified, with an epistatic effect between the two major QTL of *QGlu.spa-1A* and *QGlu.spa-1B.1* detected in four out of six environments. The peak SNPs and SNPs flanking the QTL interval of *QGlu.spa-1A* and *QGlu.spa-1B.1* were converted to Kompetitive Allele Specific PCR (KASP) markers, which can be deployed in marker-assisted breeding to increase the efficiency and accuracy of phenotypic selection for gluten strength in durum wheat. The QTL that were expressed consistently across environments are of great importance to maintain the gluten strength of Canadian durum wheat to current market standards during the genetic improvement.

## Introduction

Durum wheat (*Triticum turgidum* L. ssp. durum), a tetraploid with A and B genomes (AABB), is an economically important crop and the source of semolina for the production of pasta, couscous, and various types of baked products particularly in Mediterranean countries (Sapirstein et al., 2007). Global durum production reached 40.2 million metric tons in 2016 (http://agfax.com/2017/03/23/wheat-market-global-durum-production-expected-to-fall-in-201718/) with 7.8 million tons produced in Canada (http://www.world-grain.com/articles/news_home/World_Grain_News/2016/12/Canada_wheat_production_up_15.aspx?ID=%7BD9C6D337-5F18-480D-B635-E996394D6E6C%7D&cck=1). Gluten strength, the ability of the gluten proteins to form a satisfactory protein/starch network that promotes good cooking quality, is a key determinant of the end-use quality in durum wheat (Dexter et al., 1980). Strong gluten is a prerequisite for the production of dough with excellent rheological characteristics and hence desired quality in the finished pasta products with greater textual characteristics and increased stability to overcooking (Irvine, 1971). Gluten strength relates to the balance between viscosity and elasticity (Sissons, 2008). A positive relationship between gluten strength and low temperature dried pasta viscoelasticity has been reported (Ames et al., 2003). Strong gluten with high elastic recovery gives better cooking stability and higher cooked firmness scores (Liu et al., 1996). Rheological properties of semolina, determined by the mixograph, farinograph, extensigraph, and alveograph, are generally used to predict the cooked pasta quality (Kovacs et al., 1997). It is widely accepted that semolina from extra strong durum wheat produces firmer pasta, although the optimal level of gluten strength required for firm pasta is not clear (Sissons, 2008). Pasta quality factors of commercial importance have been the primary focus of cultivar improvement and tested for the acceptability of any new durum cultivar in Canada resulting in substantial improvement over time (Clarke et al., 2010). As such, gluten strength is an important target for genetic improvement of Canadian durum varieties.

Gluten strength variation among genotypes is mainly affected by quality and quantity of gluten proteins which are composed of polymeric glutenins and monomeric gliadins categorized by their solubility in aqueous alcohol (Autran and Feillet, 1985; Du Cros, 1987; Feillet et al., 1989; Kovacs et al., 1991; Kovacs et al., 1993). Glutenins and gliadins, together accounting for about 75–80% of total flour protein, contribute to the rheological properties of the dough (Kumar et al., 2013). Gliadins are classified as α/β, γ, and ω gliadins according to their different mobility in an acid-polyacrylamide gel electrophoresis system [reviewed by (Barak et al., 2015)]. Glutenins can be further classified into two groups based on high and low molecular weight subunits (HMW-GS and LMW-GS) reflected by their mobility during sodium dodecyl sulfate polyacrylamide gel electrophoresis (SDS-PAGE). The HMW-GS comprise about 20–30% of the glutenin (Shewry et al., 1992; Henkrar et al., 2017). LMW-GS, the major class of glutenin subunits, accounts for 70–80% of the glutenin and a strong positive correlation of LMW-GS with durum wheat quality has been reported [reviewed by (Sissons, 2008)]. The ratio of glutenin to gliadin and the ratio of HMW-GS to LMW-GS are directly related to the functional properties of the dough (Wrigley et al., 2006; Sissons et al., 2007).

Various tests were used for the prediction of gluten strength, such as SDS-sedimentation test, gluten index, alveograph, and mixograph. The SDS-sedimentation test has positive correlation with gluten strength and has been widely used for the evaluation of quality of gluten protein and for fast screening in durum wheat breeding programs due to a few advantages such as the small sample size required, simplicity, and rapidness (Dexter et al., 1980; Quick and Donnelly, 1980; Clarke et al., 1998). SDS-sedimentation volume (SV) was reported to be a good predictor of cooked pasta disk viscoelasticity (Kovacs et al., 1995a) and has been widely used for evaluation of gluten strength in durum wheat breeding programs (Clarke et al., 1998). The efficacy of SV as the predictor for gluten strength might be confounded by the low to moderate positive correlation between SV and grain protein concentration (GPC) (Kovacs et al., 1995b; Clarke et al., 2010). However, no correlation between GPC and SV was reported as well (Brites and Carrillo, 2001).

Genetic studies have proposed the quantitative nature of the gluten strength trait with multiple genes coding glutenins and gliadins. Gliadins are encoded by loci *Gli-1* and *Gli-2* located on the short arm of the homoeologous group of chromosome 1 and 6 (Payne, 1987; Anderson et al., 2009). The *Gli-B1* locus on the short arm of chromosome 1B encoding γ-gliadins bands (γ-45/γ-42) was reported to be associated with gluten strength (Joppa et al., 1983; Pasqualone et al., 2015). Selection for the favorable γ-45 gliadin allele using a monoclonal antibody was implemented in very early generation of durum breeding (Clarke et al., 1998). However, later studies indicated that it was the linked LMW-2 rather than the γ-45 gliadin that was directly associated with gluten strength (Pogna et al., 1990). LMW-GS are encoded by gene clusters at *Glu-A3* and *Glu-B3* loci tightly linked with *Gli-1* on the short arms of chromosome 1 (D’Ovidio and Masci, 2004).

The HMW-GS displayed a high level of polymorphism and are encoded by *Glu-1* loci (*Glu-A1*, *Glu-B1*) on the long arms of chromosomes 1A and 1B (Payne and Lawrence, 1983). Each *Glu-1* locus contains two closely linked genes encoding two different types of HMW-GS, higher molecular weight x-type subunit and lower molecular weight y-type subunits (Shewry et al., 1992). Not all of these *Glu-1* genes are expressed in certain cultivars, resulting in variation in HMW-GS subunit number between genotypes (Xu et al., 2009). The *Glu-B1* locus presented higher polymorphism compared with *Glu-A1*. There are considerable allelic variations at *Glu-A1* and *Glu-B1* loci and a total of 40 alleles (6 for *Glu-A1* and 34 for *Glu-B1*) and 62 subunit combinations, were detected among 205 accessions of cultivated emmer wheat (*T. turgidum* ssp. *dicoccum* Schrank) collected from different regions of Europe and China (Li et al., 2006). Similarly, a total of 43 alleles, including 5 at *Glu-A1* and 38 at *Glu-B1*, resulting in 60 different allele combinations were identified in 232 accessions of durum wheat (*T. turgidum* L. ssp. *durum*) originated from various countries (Elfatih et al., 2013).

Moreover, quantitative trait loci (QTL) associated with gluten strength of durum wheat have been reported on a number of chromosomes, including chromosomes 1 and 6. Along with the major QTL on chromosome 1B and 1AL, Blanco et al. (1998) identified six additional loci on chromosomes 3AS, 3BL, 5AL, 6AL, and 7BS associated with gluten strength. As for the most quantitative traits, it has been reported that interaction among minor QTL, and between minor QTL and environment in addition to major effect QTL determine the expression of gluten strength. Patil et al. (2009) reported three major effect QTL located on chromosome 1B in proximity to glutenin coding loci *Glu-B1*, *Glu-B2*, and *Glu-B3* along with seven epistatic QTL distributed on six chromosomes (1A, 1B, 4A, 5B, 6A, and 7A) involved in four digenic epistatic interactions (Q × Q). QTL × environment (Q × E) interactions also contributed to the variation in gluten strength (Patil et al., 2009). However, a recent study (Kumar et al., 2013) identified only one QTL consistently expressed across three environments on chromosome 1BS explaining up to 90% of the phenotypic variation and no Q x Q or Q x E interactions were observed. The differences in these studies, at least in part, could result from the different genetic background of the mapping populations. Haplotype-trait association analysis detected five loci associated with gluten index on chromosomes 1A, 1B, 2B, 4B, and 7A with the locus on 4B explaining the highest amount of phenotypic variation in 192 Canadian durum wheat breeding lines (N’Diaye et al., 2018).

Reconstituting gluten strength to current market standards during genetic improvement for other traits is difficult due to the complex quantitative nature and the environmental effect on the expression of the trait. Therefore, molecular markers closely associated with QTL underlying gluten strength are of great value for developing marker-assisted selection in the durum breeding programs. In this study, we aimed to characterize genetic components controlling gluten strength in Canadian durum wheat. Along with identification of QTL, the epistatic interaction among QTL and the interactions between QTL and environmental factors, and putative candidate genes are also reported. The findings here will facilitate the marker assisted breeding for gluten strength in durum wheat.

## Materials and Methods

### Population and Field Trials

A durum wheat population of 162 doubled haploid (DH) lines developed with the maize pollen method (Humphreys and Knox, 2015) and derived from Pelissier × Strongfield segregating for gluten strength was used in this study. Strongfield, selected from the cross AC Avonlea/DT665, is a registered Canada Western Amber Durum variety with strong gluten and low cadmium, developed at the Agriculture and Agri-Food Canada-Swift Current Research and Development Centre, Swift Current, SK (Clarke et al., 2005). Pelissier, a founder influencing the Canadian durum wheat gene pool, is a variety introduced from North Africa in 1929 (Dexter, 2008). It has high cadmium and lipoxygenase. The DH lines, along with their parents and controls were grown in field trials during year 2014, 2015, and 2016. Experiment was conducted as a randomized complete block design with two replicates at each of two seeding dates (early, E; late, L) and 1 week interval between two seeding dates per year. The field trial of each seeding date was grown at the different locations near Swift Current, SK, Canada. For phenotypic data analysis and QTL mapping, each different seeding date in each year was considered as one environment providing a total of six environments labeled as E14, L14, E15, L15, E16, and L16. Pre-plant soil testing was conducted each year to determine the rate of fertilizer application. The fertilizers were applied to target 112 kg ha^−1^ nitrogen, 67 kg ha^−1^ phosphorus, and 22 kg ha^−1^ sulfur. The soil is naturally high in potassium and did not require additional application.

### Gluten Strength Measurement

The seeds harvested from each replicate of each seeding date were subjected to gluten strength measurement. Therefore, a total of four replicates of samples from each year/location over 3 years were analyzed. The durum whole grain samples were ground on an Udy mill with 1-mm screen at 13% moisture basis. The gluten strength was determined on 2.5 g samples of whole grain flour samples using the SDS-sedimentation volume (SV) method of Dick and Quick (1983) as modified by Agriculture and Agri-Food Canada (AAFC) with the addition of 25 ml of distilled water and 25 ml SDS solution to each sample. The higher the SV, the stronger the gluten.

### Statistical Analysis and Biplot Analysis of Genotype-by-Environment Interaction

Pairwise phenotypic correlations were calculated using the Pearson’s correlation coefficient in the R package Hmisc (version 4.2-0, http://cran.r-project.org/web/packages/Hmisc/index.html).

Analysis of variance (ANOVA) was performed using the PROC MIXED procedure of SAS 9.3 (SAS Institute, Cary, NC, USA). In the mixed model, lines were considered as fixed effects, and years, seeding dates, line × year interactions, line × seeding date interactions, line × year × seeding date interactions, seeding dates nested in years, and replications nested in years and seeding dates were considered as random effects. The heritability of SV was calculated as the ratio of the genetic variance and the phenotypic variance across years using σ_g_^2^/(σ_g_^2^ + σ_gy_^2^/y + σ_gs_^2^/s + σ_gys_^2^/ys + σ_ϵ_^2^/rys), where σ_g_^2^, σ_gy_^2^, σ_gs_^2,^ σ_gys_^2^, and σ_ϵ_^2^ were estimates of line, line × year interaction, line × seeding date interaction, line × year × seeding date interaction, and residual variance, respectively, and y, s, and r represented the numbers of year, seeding date, and replication, respectively, The repeatability of SV was calculated as the ratio of the genetic variance and the phenotypic variance of individual year using σ_g_^2^/(σ_g_^2^ + σ_gs_^2^/s + σ_ϵ_^2^/rs), where σ_g_^2^, σ_gs_^2^, and σ_ϵ_^2^ were estimates of line, line × seeding date interaction and residual variance, respectively, and s and r represented the numbers of seeding date and replication. For the estimations of the heritability and repeatability, all effects were considered random.

Biplot analysis of genotype-by-environment interaction was performed with the GGEBiplotGUI R (R version 3.0.3) package (Frutos et al., 2014). The analysis was based on a “tester-centered (G + GE)” table and row metric preserving, without any scaling.

### Genotyping and Genetic Map Construction

DNA was extracted from leaves of 2-week-old seedlings of DH lines and parents using the AutoGenprep 965 (AutoGen Inc, Holliston, MA). The Infinium iSelect Wheat 90K SNP chip was used for genotyping according to the manufacturer’s protocols (Illumina). Single nucleotide polymorphism (SNP) allele clustering and genotype calling was performed with GenomeStudio v2011.1 as described by Cavanagh et al. (2013). The default clustering algorithm implemented in GenomeStudio was first used to identify assays that produced three distinct clusters expected for bi-allelic SNPs. Manual curation was performed for assays that produced compressed SNP allele clusters that could not be discriminated by the default algorithm. The accuracy and robustness of SNP clustering was visually validated. SNPs with poor clustering quality, more than 30% missing data, or segregation distortion of more than 0.35 were removed. Redundant SNPs were also removed in R/qtl (Broman et al., 2003).

A total of 1,212 polymorphic SNP markers were used for genetic map construction in MapDisto version 2.0 software (Heffelfinger et al., 2017). Markers were classified into linkage groups based on a logarithm of odds (LOD) score threshold of 7.0 and recombination of 0.3. Genetic distances in cM were estimated using Kosambi’s mapping function. Markers within each group were ordered using the AutoOrder command with the Seriation II method. The marker order was refined using CheckInversion and Ripple command with the sum of adjacent recombination frequencies (SARF) option. Markers showing double recombination events were re-scored. Markers detected with genotyping errors were replaced by missing values. All calculations were repeated for new linkage groups. The markers were distributed over 25 linkage groups (LGs). LGs were assigned to chromosomes based on comparison with an existing high-density SNP-based consensus map of durum wheat (Maccaferri et al., 2015). Parents were genotyped with the published molecular markers that discriminate glutenin and gliadin to test if they are polymorphic at these loci and to facilitate the comparative mapping.

### Quantitative Trait Locus Mapping

Each different seeding date in each year was considered as one environment. Mean values for the trait from two replicates in each environment were used for the detection of QTL. Outliers of trait values were detected and removed using a Z-score transformation with a threshold of 3. QTL detection was performed using composite interval mapping (CIM) in WinQTL Cartographer v.2.5 software (http://statgen.ncsu.edu/qtlcart/WQTLCart.htm; Wang et al., 2012). A walking speed of 1 cM was used. Forward regression was used for the selection of the markers to control the genetic background (control markers or cofactors) with up to five control markers. A window size of 10 cM was used to exclude closely linked control markers at the testing site. The LOD threshold at a significance level of 0.05 for declaring statistically significant QTL was calculated by 1,000 permutations. The additive effect (a) and phenotypic variance explained by each QTL (*R* ^2^) were estimated by CIM. The identified QTL (LOD > threshold) were automatically localized with the following parameters: minimal space between peaks = 30 cM; and minimum LOD from top to valley = 1.4. QTL detected in different environments were considered to be the same if the confidence intervals overlapped and the trait enhancing allele was contributed by the same parent.

The digenic epistatic interactions among all pairwise combinations of QTL were analyzed with multiple interval mapping (MIM) in the WinQTL Cartographer v.2.5 software. The initial QTL model was set using the CIM results obtained in each environment. The QTL model was progressively refined by searching and testing QTL or epistasis, and re-estimating. Both main additive effects of QTL and their epistatic interactions were tested for significance using the Bayesian information criterion (BIC). Not only main QTL (QTL with statistically significant main effect) and interactions among main QTL, but epistatic QTL (QTL that has no or small main effect but statistically significant interaction effect with another QTL) interacting with the main QTL were searched.

Multiple-trait composite interval mapping (Mt-CIM) implemented in WinQTL Cartographer v.2.5 was used to test for the presence of Q × E interaction at the main chromosome regions affecting the target trait (Maccaferri et al., 2008; Wang et al., 2012). The value of the trait in each environment was treated as a separate trait for the common genotypes. The G × E (H4) hypothesis was tested. All reported QTL were designated according to the Recommended Rules for Gene Symbolization in Wheat (http://wheat.pw.usda.gov).

### Comparative Mapping and Projection of Quantitative Trait Locus Markers Onto the Durum Wheat Consensus Genetic Map and Onto the Reference Genomes of Durum and Wild Emmer Wheat

QTL reported in the literature and identified in this study were projected onto the durum high-density consensus genetic map developed by Maccaferri et al. (2015) which includes SNP, simple sequence repeat (SSR), and diversity array technology (DArT) markers by projecting either a single marker near the QTL peak position or a pair of flanking markers within the QTL interval. The genetic linkage map and the QTLs were drawn using MapChart (version 2.3) software (Voorrips, 2002). Pairwise Spearman’s rank correlation was performed in R version 3.3.2 to compare the collinearity of the marker order on the chromosomes of the durum consensus map and the genetic map generated in this study.

The sequences of the 90K SNPs were downloaded from the Kansas State University SNP marker database (http://wheatgenomics.plantpath.ksu.edu/snp/). Sequences of SSR markers were retrieved from the GrainGenes database (https://wheat.pw.usda.gov/GG3/). Sequences of DArT markers were downloaded from https://www.diversityarrays.com/technology-and-resources/sequences. Physical map positions of SNP, SSR, and DArT markers on genomes of durum wheat cv. Svevo (Maccaferri et al., 2019) and wild emmer wheat accession Zavitan (Avni et al., 2017) were aligned using the BLAST from Durum Wheat Genome Database (http://d-data.interomics.eu) and GrainGenes database (https://wheat.pw.usda.gov/GG3/wildemmer_blast). QTL markers on the physical map of durum wheat cv. Svevo and wild emmer wheat accession Zavitan were drawn with PhenoGram software (http://visualization.ritchielab.org/phenograms/plot).

### Development of Kompetitive Allele Specific PCR Markers

Firstly, several SNPs in the interval of each of the target QTL were tested for 22 DH lines plus parents using the Kompetitive Allele Specific PCR (KASP) primers available for the Infinium iSelect Wheat 90K SNP chip (http://www.polymarker.info/designed_primers). Then, two closest KASP markers to each target QTL were used to genotype the population. The KASP assays were performed as described by Rasheed et al. (2016).

## Results

### Phenotypic Variation Among Doubled Haploid Population

**Figure 1:**
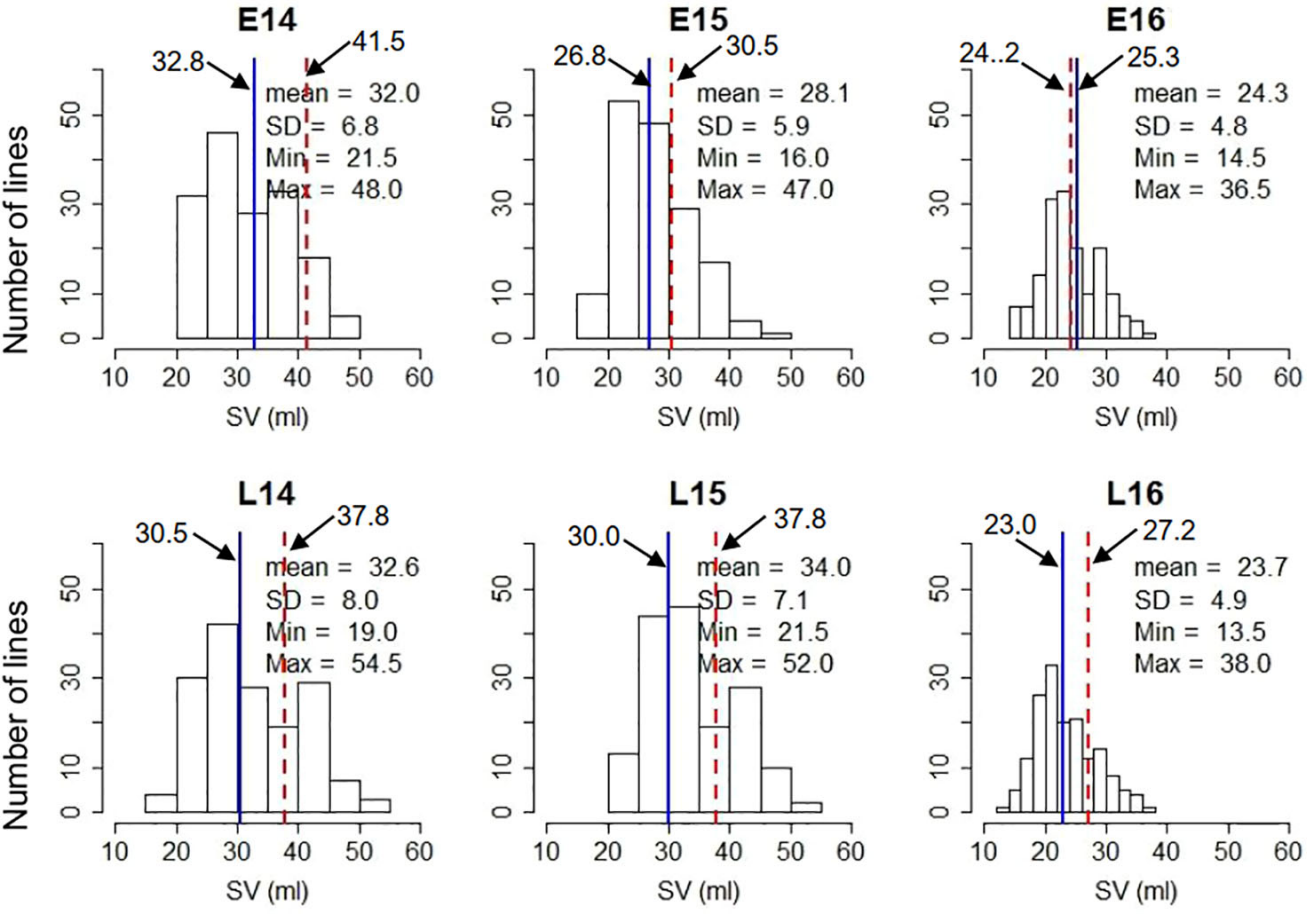
Frequency distribution of SDS-sedimentation volume (SV) in the Pelissier × Strongfield population from 2014 to 2016 field trials with two seeding dates in each year and two replicates at each seeding date. Top panel, early seeding date; bottom panel, late seeding date. The blue solid line represents Pelissier; the red dashed line represents Strongfield; the mean SV of parents in each seeding date were shown; SD, standard deviation; Max, maximum; Min, minimum.

The gluten strength of the DH population was measured using SV. The summary statistics including mean SV values, range [minimum and maximum values, and standard deviation (SD)] are shown in **Figure 1**. Strongfield had significant higher SV value than Pelissier in all environments except E15 and E16 (**Figure 1** and **Table S1**). The population had the highest mean value in environment L15 (mean = 34.0) and the lowest in L16 (mean = 23.6) which reflects the environmental effect on gluten strength. Nevertheless, except in year 2015, no significant difference was observed for the mean SV of the population between two seeding dates. Although seeding date had no significant effect, the interaction of line by year and by seeding date was significant (**Table S2**). SV showed high Pearson’s correlations among DH lines across environments ranging from *r *= 0.85 to 0.92 (**Figure S1**). The population had the largest phenotypic variation in environment L14, as indicated by the highest standard deviation (SD) and coefficient of variation (CV), and the least variation in environment E16. Individual DH lines displayed bi-directional transgressive segregation, as shown by the maximum and minimum values relative to the parents (**Figure 1**). The transgressive segregation might result from the recombination of favorable or deleterious additive alleles from the parents, epistatic interactions of two genes, or any combinations of these mechanisms. The lines carrying favorable alleles from both parents showed higher SV than parent Strongfield, while the lines with the trait decreasing alleles from both parents had lower SV than Pelissier.

**Figure 2:**
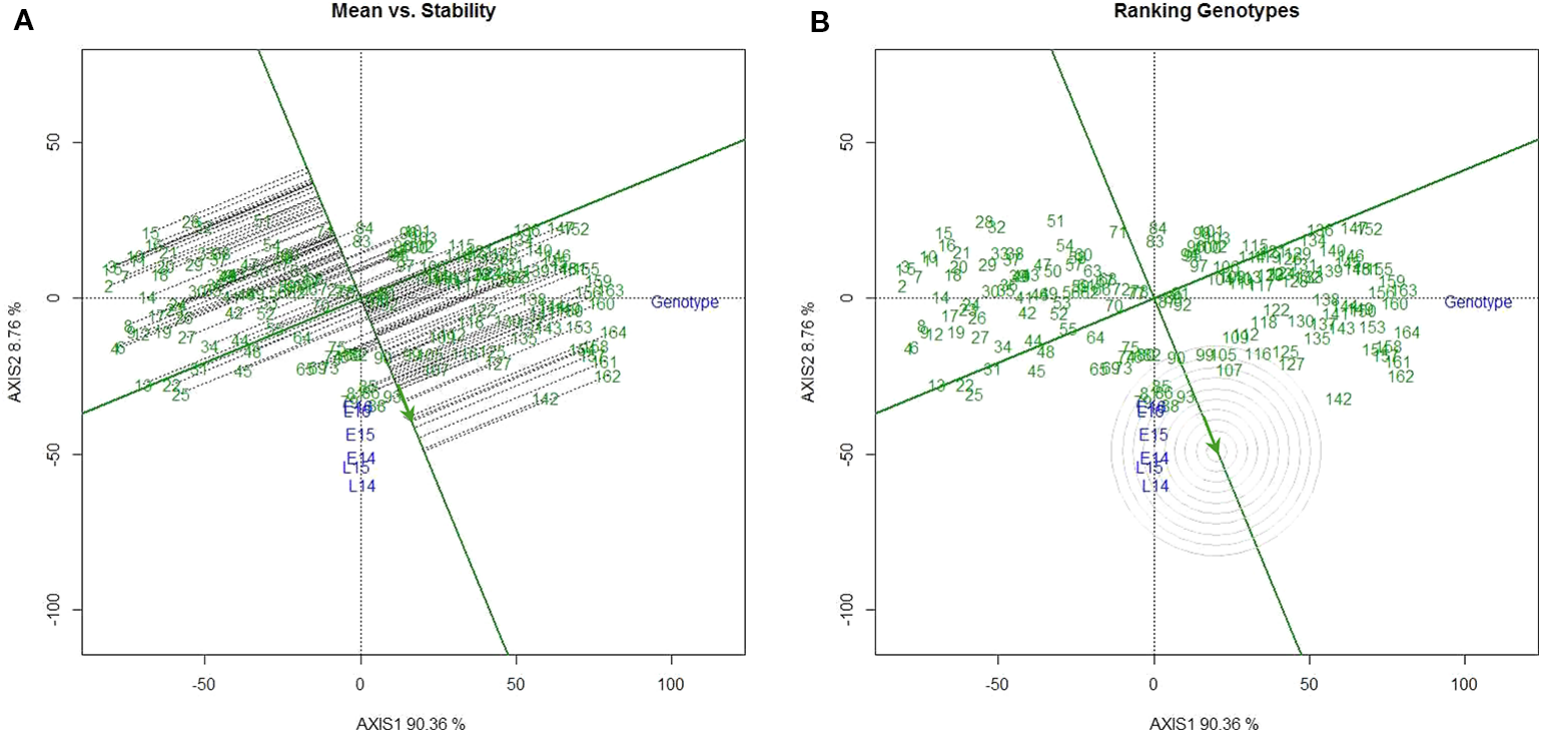
GGE (Genotypic main effect plus Genotype-by-Environment interaction) analysis of SDS-sedimentation volume (SV) in DH lines of Pelissier × Strongfield. **(A)** Average-environment coordination (AEC) view of the GGE biplot; the single-arrowed line is the AEC abscissa (or AEA) and points to the higher mean SV value across environments. **(B)** Ranking doubled haploid (DH) lines relative to the ideal genotype (a genotype that GGE predicted has the best performance across environments for SV) on SV performance. The arrow is where an ideal genotype should be. The DH lines located closer to the ideal genotype are more desirable than others.

The percentages of GGE (Genotypic main effect plus Genotype-by-Environment interaction) explained by the first principal component was 90.4% and second principal component was 8.8% (**Figures 2A, B**). The DH lines were ranked based on both mean performance and stability across environments. The single arrowed line in **Figure 2A** points to higher mean SV value across environments. Therefore, line 162 had the highest mean SV value, while line 15 had the lowest mean SV value. The AEC (average-environment coordination) ordinate (dashed line) points to the greater variability (poor stability) in either direction. Thus, lines 90 and 93 were the most stable lines across environments (**Figure 2A**). The position of the ideal genotype which has the highest performance in all environments, is indicated by the arrow in **Figure 2B**. The DH lines located closer to the ideal genotype are more desirable than others. Taken into account both mean SV and stability, line 93 was the most desirable genotype.

Significant weak to moderate positive correlation between SV and GPC was observed in three out of six environments (*r *= 0.3–0.36) in this population (**Figure S2A**). In year 2014, significant correlation was shown in both seeding dates when the population had lower GPC compared to other field years. No significant correlation existed for any seeding date in year 2015. Similarly, significant weak to moderate negative correlation between SV and grain yield (GY) was also observed in the same three out of six environments [*r *= −0.334–(−0.216)] (**Figure S2B**). Significant negative correlation was displayed in both seeding dates in year 2014 when higher GY was obtained. While no significant correlation was observed in the year 2015 with lower GY.

### Quantitative Trait Locus Mapping by Composite Interval Mapping in Single Environments

ANOVA (**Table S2**) indicates that genotype by year and by seeding date interaction had significant effect on the SV. Therefore, QTL analysis was first performed for SV in each environment. Variable numbers of significant QTL, from two to five, were detected in each environment. Globally, the largest number of QTL (5) was detected in environment L14 and L15. A total of nine different QTL were detected across environments, four of which were specific for a single environment (**Table 1**). Both parental lines contributed the favorable alleles depending on the QTL (2 by Strongfield and 7 by Pelissier). A major QTL on chromosome 1B (*QGlu.spa-1B.1*) explaining up to 40.1% of the phenotypic variance (*R^2^*) and a second major QTL on chromosome 1A (*QGlu.spa-1A*) explaining up to 18.7% of the phenotypic variance, were detected across all environments with high SV allele derived from Strongfield. Two minor QTL, *QGlu.spa-1B.2* and *QGlu.spa-1B.3* with *R^2^* values of 4.1% and 6.3%, were also detected on chromosome 1B but only in a single environment. Additionally, three QTL on chromosome 2B and two QTL on chromosome 3A were detected with *R^2^* values ranging from 3.4 to 8.9%. The QTL *QGlu.spa-3A.1* and *QGlu.spa-3A.2* were repeatedly detected in at least two environments. Except *QGlu.spa-1A* and *QGlu.spa-1B.1*, no other minor QTL was detected in L16. Pelissier contributed trait-enhancing alleles to all minor QTL.

**Table 1:** Overview of quantitative trait loci (QTL) identified for SDS-sedimentation volume (SV) across six environments.

Chr^a^	QTL	Env^b^	Peak marker	LOD	Additive^c^	*R^2^*(%)^d^	Interval (two LOD drop)
1A	*QGlu.spa-1A*	E14	wsnp_Ex_c13186_20822127	16.1	3.00	18.7	BS00088136_51 - Kukri_c10405_1277
		E15	wsnp_Ex_c13186_20822127	15.1	2.49	17.6	IAAV1142 - RAC875_c31031_387
		E16	wsnp_Ex_c13186_20822127	10.7	1.83	13.7	IAAV1142 - RAC875_c31031_387
		L14	wsnp_Ex_c13186_20822127	14.3	3.09	14.6	BS00088136_51 - RAC875_c31031_387
		L15	wsnp_Ex_c13186_20822127	18.1	3.14	18.2	IAAV1142 - RAC875_c31031_387
	**	L16	wsnp_Ex_c13186_20822127	10.8	2.03	16.9	IAAV1142 - RAC875_c31031_387
1B	*QGlu.spa-1B.1*	E14	Kukri_c38353_67	26.5	4.11	35.6	BS00085235_51 - RCA875_rep_c74067_541
		E15	Kukri_c38353_67	25.6	3.48	34.6	BS00085235_51 - RCA875_rep_c74067_541
		E16	Kukri_c38353_67	27.2	3.01	35.1	BS00085235_51 - RCA875_rep_c74067_541
		L14	Kukri_c38353_67	22.2	4.71	25.4	BS00085235_51 - RCA875_rep_c74067_541
		L15	Kukri_c38353_67	32.7	4.60	40.1	BS00085235_51 - RCA875_rep_c74067_541
		L16	Kukri_c38353_67	20.4	2.98	36.5	BS00085235_51 - RCA875_rep_c74067_541
	*QGlu.spa-1B.2*	L14	Excalibur_c50079_420	5.4	-1.80	4.1	Ku_c241_460 - BS00078029_51
	*QGlu.spa-1B.3*	L15	BS00067436_51	7.4	-1.91	6.3	Tdurum_contig7449_800 - RAC875_c47427_235
2B	*QGlu.spa-2B.1*	L15	RAC875_c38003_164	4.2	-1.37	3.4	Excalibur_c19499_948 - D_F5XZDLF01CFO7W_135
	*QGlu.spa-2B.2*	E16	Kukri_c25868_56	7.3	-1.44	8.7	Kukri_c25868_56 - Ex_c55735_1012
	*QGlu.spa-2B.3*	L14	Excalibur_c91034_141	5.5	-1.79	5.6	Excalibur_c33221_681 - CAP7_6910_523
3A	*QGlu.spa-3A.1*	E14	RAC875_c64107_404	4.1	-1.41	4.0	RAC875_c64107_404 - BS00021981_51
		L15	RAC875_c64107_404	4.4	-1.40	3.6	RAC875_c64107_404 - BS00021981_51
	*QGlu.spa-3A.2*	E15	Excalibur_c14216_692	5.6	-1.46	5.9	Tdurum_contig98188_239 - RAC875_c775_1264
		E16	wsnp_Ex_rep_c69864_68824236	6.7	-1.42	8.3	CAP_c3367_68 - Tdurum_contig98188_239
		L14	Excalibur_c14216_692	9.7	-2.45	8.9	Tdurum_contig98188_239 - RAC875_c775_1264

^a^Chromosome; ^b^environment.

^c^Additive effect, the positive values indicate that the alleles from Strongfield have the effect of increasing the trait value.

^d^R^2^ is the percentage of phenotypic variation explained by each QTL.

### Multi-Environment Quantitative Trait Loci Analysis

**Figure 3:**
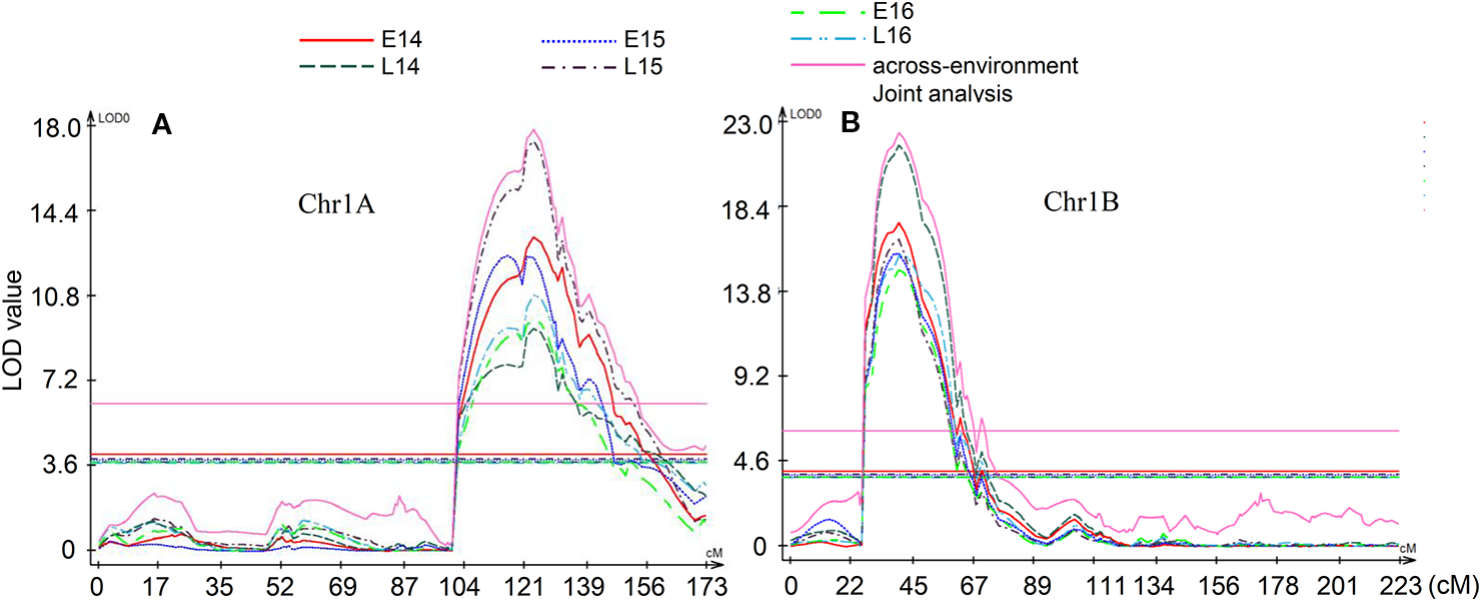
Multi-environment quantitative trait loci (QTL) analysis. **(A)** QTL *QGlu.spa-1A* on chromosome 1A, **(B)** QTL *QGlu.spa-1B.1* on chromosome 1B. The horizontal lines indicate logarithm of odds (LOD) significance threshold determined by 1,000 permutations at a significance level of 0.05.

Multi-environment QTL analysis was performed to detect the significant QTL across environments and Q × E effect (**Figure 3**). Two significant QTL *QGlu.spa-1A* and *QGlu.spa-1B.1* were detected by multi-environment analysis which agreed with single-environment analysis. In these analyses, environment L15 influenced joint analysis the most for QTL *QGlu.spa-1A* while environment L14 influenced joint analysis the most for QTL *QGlu.spa-1B.1*. However, environment L14 influenced SV the least for QTL *QGlu.spa-1A*. Both *QGlu.spa-1A* and *QGlu.spa-1B.1* were statistically significant for multi-environment joint analysis. Although the Q × E effect was significant, these two QTL were stably expressed across all environments. The QTL mapped on other chromosomes in only one or two environments did not reach the significance threshold value in the multi-environment QTL analysis. Notably, the Q × E effect observed was due to the difference of the effect in magnitude and not the direction of QTL. This was also evidenced by the consistent sign of the effects of QTL detected across environments (**Table 1**).

### Implication of Quantitative Trait Loci on 1A and 1B and Development of Kompetitive Allele Specific PCR Markers

**Figure 4:**
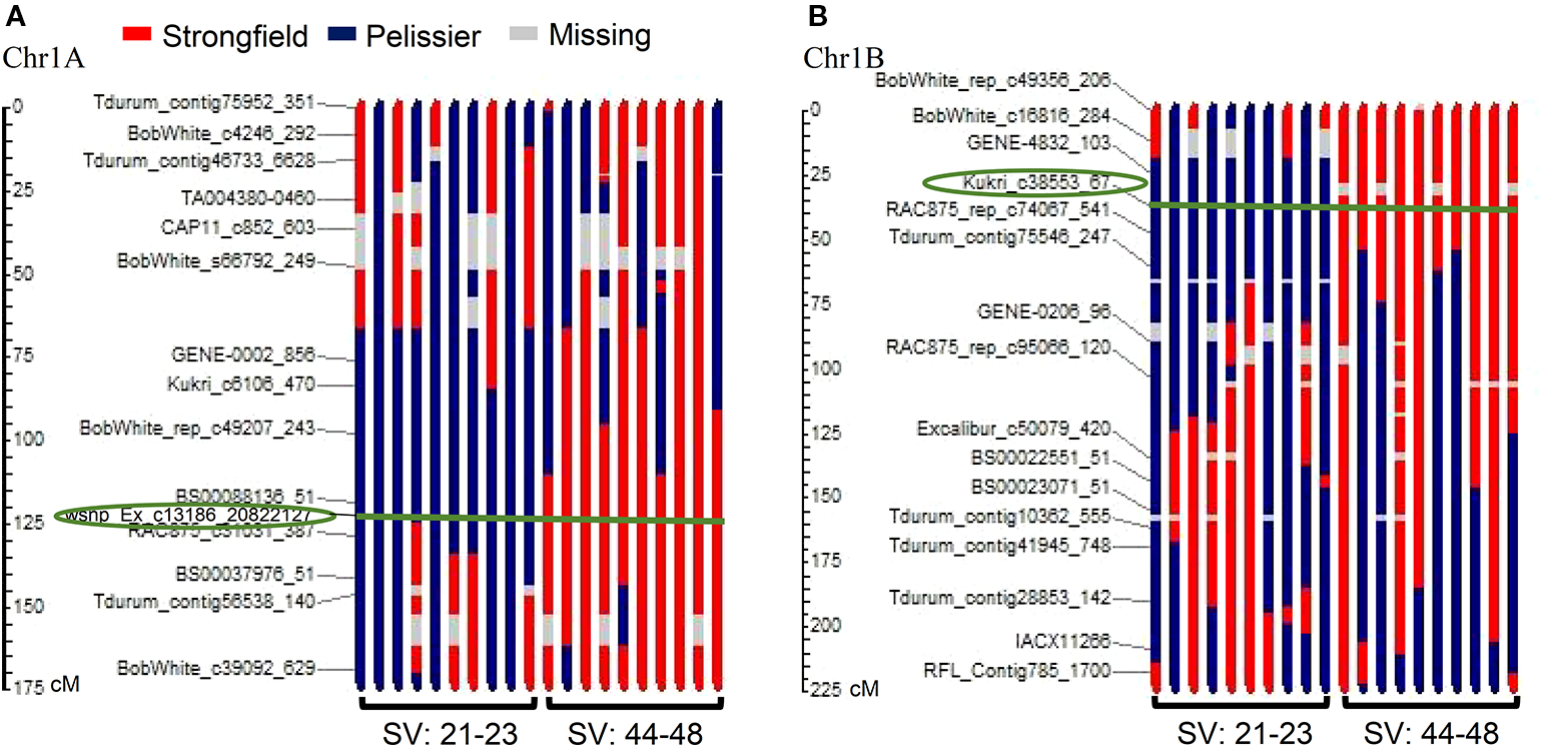
Graphical illustration of genotypes of 20 doubled haploid (DH) lines with recombination pattern of major quantitative trait loci (QTL) **(A)** *QGlu.spa-1A* on chromosome 1A and **(B)** *QGlu.spa-1B.1* on chromosome 1B. Circled marker is the peak marker in the QTL region and the green line indicates the peak marker position on each genotype. The blue bar represents the fragment derived from weak gluten strength parent Pelissier and the red bar represents the fragment derived from strong gluten strength parent Strongfield. SV, SDS-sedimentation volume.

**Figure 5:**
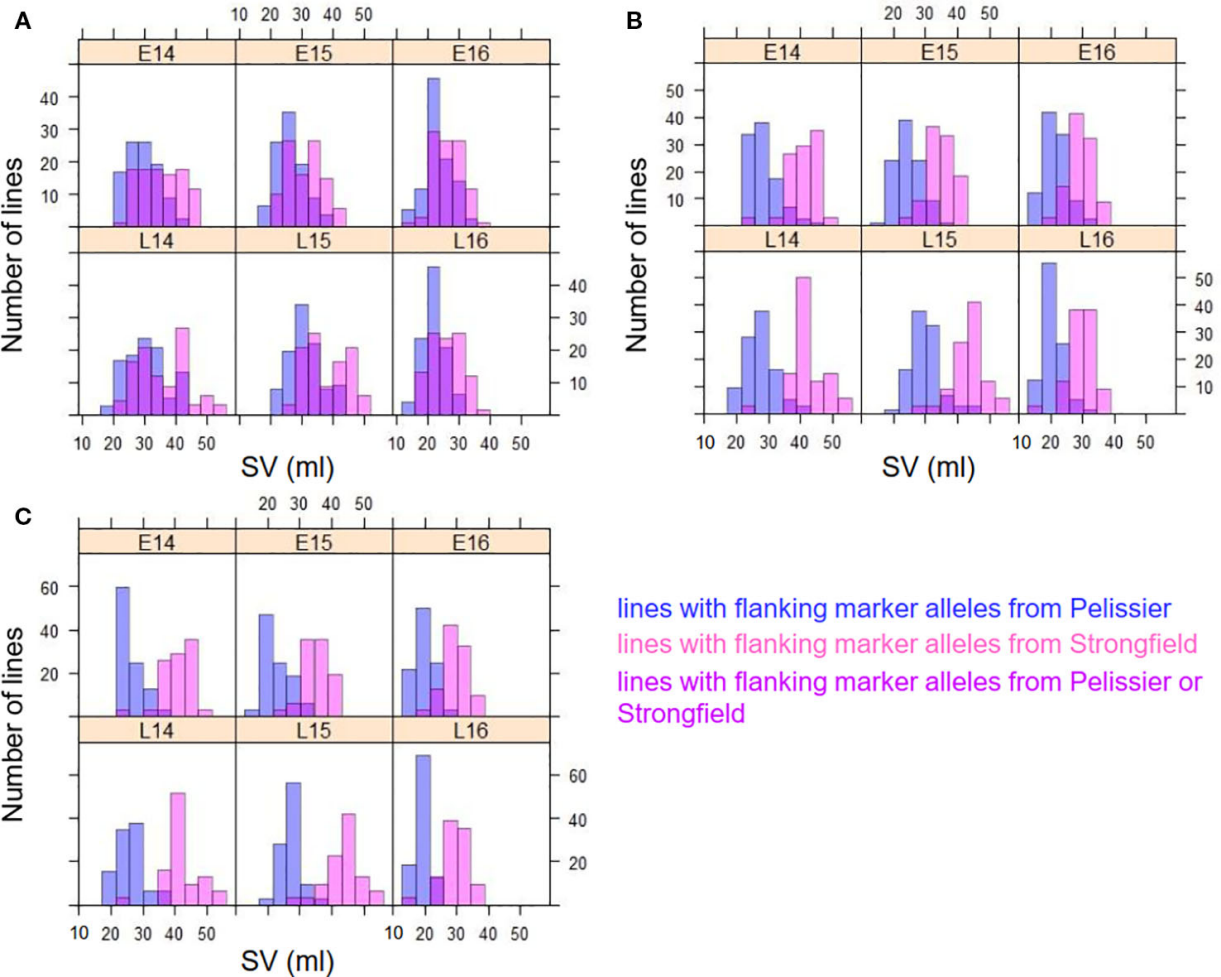
Frequency distribution of SDS-sedimentation volume (SV) in two groups of DH lines separated on the genotype of two flanking markers of **(A)** *QGlu.spa-1A* (IAAV1142 and RAC875_c31031_387) on chromosome 1A, **(B)** *QGlu.spa-1B.1* (Kukri c38553_67 and RCA875_rep_c74067_541) on chromosome 1B, and **(C)** both *QGlu.spa-1A* on chromosome 1A and *QGlu.spa-1B.1* on chromosome 1B across six environments (field year 2014–2016 with two seeding dates in each year. early, E; late, L). The blue bar represents the lines with alleles from weak gluten strength parent Pelissier, the pink bar represents the lines with alleles from strong gluten strength parent Strongfield, and the magenta bar represents the lines with alleles either from Strongfield or Pelissier.

**Figure 4A** is a graphical illustration of the chromosome 1A region harboring QTL *QGlu.spa-1A* of a selection of 20 DH genotypes and **Figure 4B** of the chromosome 1B region containing *QGlu.spa-1B.1* for the same genotypes with high and low SV values. On both chromosome 1A and 1B, the Strongfield alleles occurred in high SV genotypes, whereas the Pelissier alleles contributed to the low SV lines. This agreed with QTL analysis results (**Table 1**). Colored fragments along the chromosome region outside the green line of the peak marker, refer to the loci belonging to other traits may be not related to SV. Based on the genotypes of two flanking markers in the QTL region of *QGlu.spa-1A* (IAAV1142 and RAC875_c31031_387) and *QGlu.spa-1B.1* (Kukri c38553_67 and RCA875_rep_c74067_541), the DH lines were separated into two groups with significantly different means (*t* test, *p <*10^−4^) between the two groups (**Figures 5A, B**). More distinct separation was shown for the *QGlu.spa-1B.1* than *QGlu.spa-1A*, which is in agreement with a larger portion of the phenotypic variation explained by *QGlu.spa-1B.1* (**Table 1**). Based on the genotypes of the flanking markers of both aforementioned QTL combined, two main groups with clearer separation was observed within the population: one group of DH lines with high SV value (strong gluten) having flanking marker alleles from Strongfield and the other group of lines with low SV value (weak gluten) carrying flanking marker alleles from Pelissier (**Figure 5C**).

**Figure 6:**
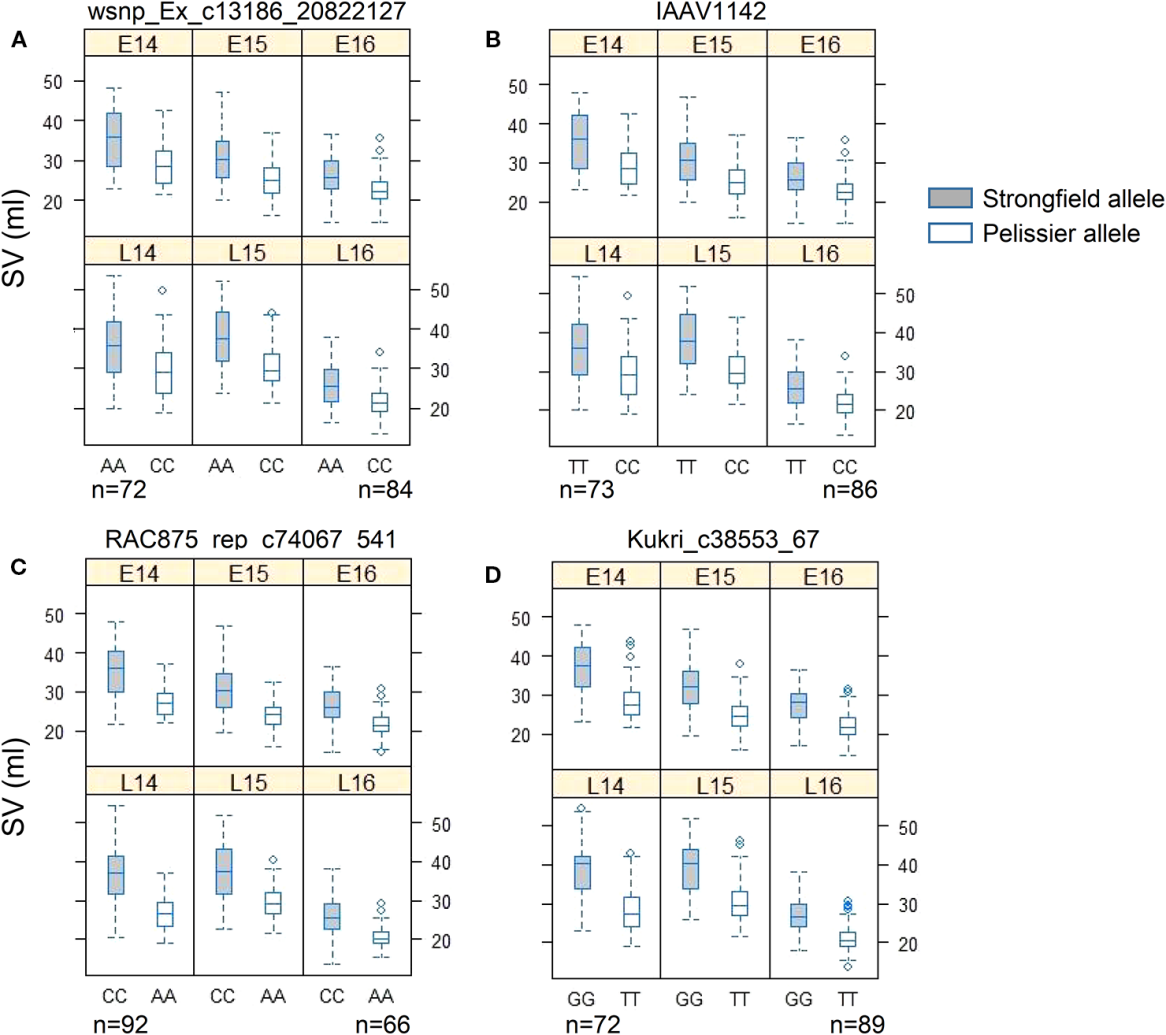
Phenotypic validation of Kompetitive Allele Specific PCR (KASP) assays for **(A)** wsnp_Ex_c13186_20822127 and **(B)** IAAV1142 in the interval of *QGlu.spa-1A* on chromosome 1A, **(C)** RAC875_rep_c74067_541, and **(D)** Kukri_c38553_67 in the interval of *QGlu.spa-1B.1* on chromosome 1B across six environments (field year 2014–2016 with two seeding dates in each year. early, E; late, L). The *p* value of *t* test of two genotype groups for each marker in each environment is smaller than 0.001. SV, SDS-sedimentation volume.

Two KASP assays were developed for each of QTL, *QGlu.spa-1A* (wsnp_Ex_c13186_20822127 and IAAV1142), and *QGlu.spa-1B.1* (RAC875_rep_c74067_541 and Kukri_c38553_67). All KASP assays were validated against the SV values of the population in each environment (**Figure 6**). In all cases, the genotypes carrying Strongfield allele had significantly higher SV than those with Pelissier allele.

### Combined Haplotype Analysis Across Multiple Quantitative Trait Loci

**Figure 7:**
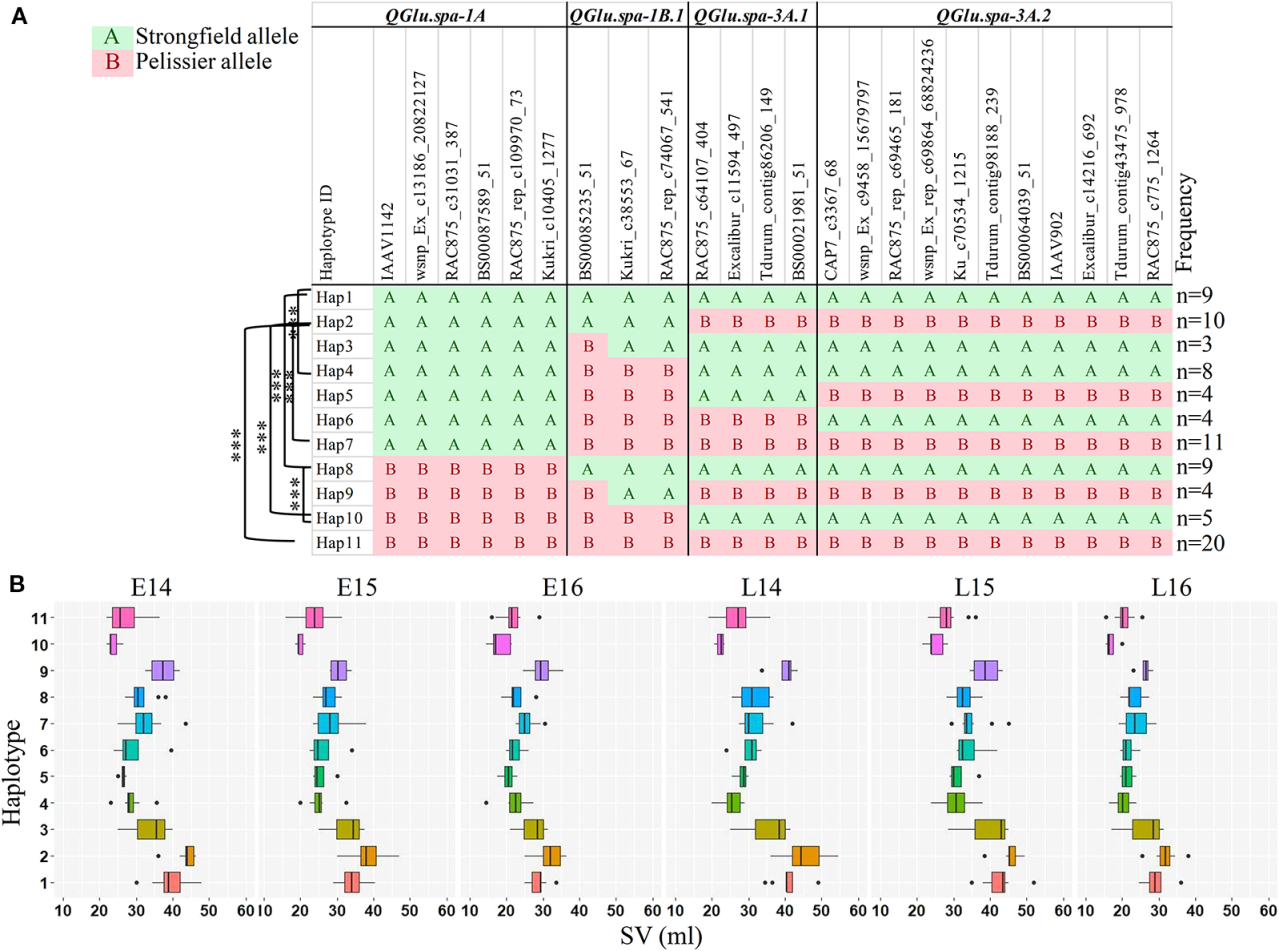
Haplotype analysis across four quantitative trait loci (QTL) [two logarithm of odds (LOD) interval] which were identified in at least two environments. **(A)** Haplotype block based on single nucleotide polymorphism (SNP) markers in each QTL region. **(B)** Boxplots of the phenotype values corresponding to 11 different haplotype groups in each environment. Haplotypes containing less than three doubled haploid (DH) lines were omitted from the table. The DH lines with undetermined haplotype were not shown. Haplotypes were assigned using R package Haplotyper. ***, significant at *p* < 0.001 (*t* test). SV, SDS-sedimentation volume.

To investigate the accumulated effects of the favorable alleles on SV across multiple QTL, the combined haplotype analysis was performed on QTL detected in two or more environments, *QGlu.spa-1A*, *QGlu.spa-1B.1*, *QGlu.spa-3A.1*, and *QGlu.spa-3A.2*. The SNPs in the two LOD interval of each QTL were used for haplotype analysis. A total of 11 different haplotypes (Hap1–Hap11) were identified at different frequencies, with each haplotype containing three or more DH lines (**Figure 7**). The DH lines with Hap2 has the best combination of all favorable alleles at each QTL, as evidenced by the highest mean SV across all environments. The most desirable genotype, line 93, has this haplotype. While the lines with Hap10 has the least favorable combination of the alleles from each QTL. Significant difference was observed for SV in these two haplotype groups across all environments. Significant difference in SV between Hap1 and Hap8 across all environments agreed with the effect of the *QGlu.spa-1A.* Likewise, the significant difference in SV between Hap1 and Hap4, Hap2 and Hap7, Hap8 and Hap10, confirmed the effect of major QTL *QGlu.spa-1B.1*. Except in E16, no significant difference was observed between Hap1 and Hap2. This is not surprising given the environment specific expression and minor effect of *QGlu.spa-3A.1* and *QGlu.spa-3A.2.*

### Identification of Epistatic Interaction of Quantitative Trait Loci

Multiple interval mapping (MIM) has been used for mapping multiple QTL with epistasis (Laurie et al., 2014). In this study MIM was used for identification of digenic epistatic interactions among all pairwise combinations of QTL. Compared with the results obtained by CIM analysis, two additional significant QTL, *QGlu.spa-1B.4* in L14, and *QGlu.spa-5A* in L15, were detected with *R* ***^2^*** values of 9.4% and 2.3% respectively (**Table 2**). The epistatic interactions detected together with their average effects and *R^2^* values are reported in **Table 2**. A total of 11 pairwise QTL interactions (additive × additive) were detected in different environments at a significance level of 0.05. Of note, the epistatic effect between the two major QTL *QGlu.spa-1A* and *QGlu.spa-1B.1* was detected in four out of six environments with *R^2^* values of 1.4–1.9%. The other 10 pairs of environment-specific interactions with *R^2^* values of 0.8–3.7% were detected only in a single environment. Not only interactions among main QTL (QTL with statistically significant main effect) but also epistatic QTL, QTL that has no or small main additive effect but statistically significant interaction effects with another QTL, interacting with main QTL were identified. It is interesting to note that the additive effect of *QGlu.spa-5B was* too small to reach the genome-wide significance level in CIM scans but it had significant interaction with identified QTL *QGlu.spa-1B.4* and *QGlu.spa-2B.3* in environment L14, as well as with QTL *QGlu.spa-2B.4* in L16. Likewise, epistatic QTL *QGlu.spa-6B* had significant interaction with other QTL in two environments, E14 and E16, but no main effect.

**Table 2:** Epistatic interaction between quantitative trait loci (QTL).

QTL1^a^	QTL2	Environment	LOD	Effect^b^	*R^2^* (%)^c^	Empirical *p*-Value
*QGlu.spa-1A*	*QGlu.spa-1B.1*	E14	3.0	2.3	1.5	0.002
		E15	2.1	1.8	1.8	0.015
		L14	3.6	2.3	1.4	<0.0001
		L16	2.2	1.9	1.9	0.028
*QGlu.spa-1A*	*QGlu.spa-1B.2*	L16	3.2	3.5	3.0	<0.0001
*QGlu.spa-1A*	*QGlu.spa-1B.3*	L15	1.6	1.8	0.8	0.022
*QGlu.spa-5A*		E16	4.2	4.6	2.2	<0.0001
		L15	2.9	2.7	2.3	0.001
*QGlu.spa-1B.1*	*QGlu.spa-5A*	L15	1.8	1.8	1.0	0.02
*QGlu.spa-1B.1*	QGlu.spa-6B*	E14	1.7	-1.7	1.2	0.036
*QGlu.spa-1B.4*		L14	7.1	2.0	9.4	<0.0001
*QGlu.spa-1B.4*	QGlu.spa-5B*	L14	2.8	-2.0	1.5	<0.0001
QGlu.spa-6B*		E14	0.3	0.4	0.5	0.777
		E16	0.5	0.6	0.5	0.477
*QGlu.spa-2B.3*	QGlu.spa-5B*	L14	5.0	6.5	1.8	<0.0001
*QGlu.spa-2B.3*	QGlu.spa-6B*	E16	3.6	4.7	2.2	<0.0001
QGlu.spa-2B.3*	QGlu.spa-2B.4*	L16	3.4	3.2	1.4	<0.0001
QGlu.spa-2B.4*	*QGlu.spa-3A.2*	L16	2.8	3.4	3.7	<0.0001
QGlu.spa-2B.4*	QGlu.spa-5B*	L16	3.0	3.6	2.0	<0.0001

a QTL1 and QTL2 are a pair of interacting QTL.

b Epistatic effect of QTL1 and QTL2.

c R^2^ is the percentage of phenotypic variation explained by QTL or QTL epistasis.

*Epistatic QTL, QTL that has no or small main additive effect but statistically significant interaction effect with another QTL.

### Comparison With Previously Reported Quantitative Trait Loci

**Figure 8:**
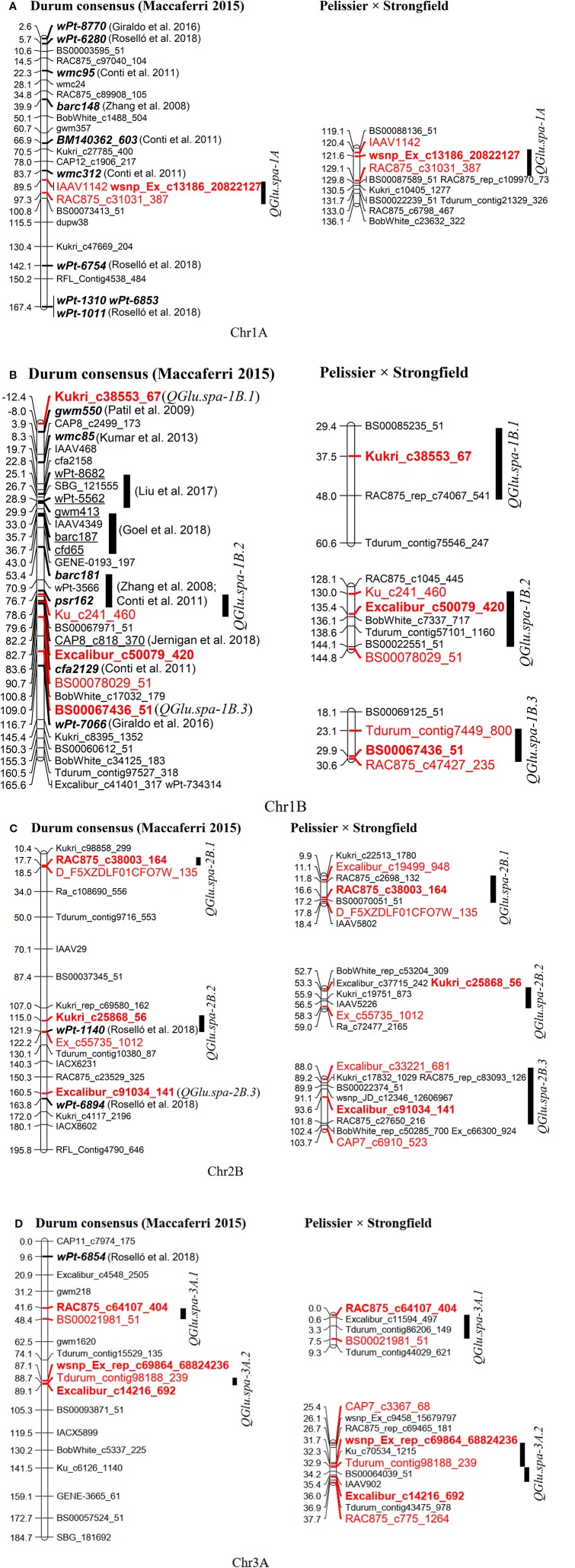
Projection of quantitative trait loci (QTL) for SDS-sedimentation volume (SV) reported in the literature (in both bread wheat and durum wheat) and QTL identified in this study onto the durum wheat consensus genetic map developed by Maccaferri et al. (2015) (left). The QTL on the genetic map from this present study is shown on the right. **(A)** Chromosome 1A, **(B)** 1B, **(C)** 2B, and **(D)** 3A. The markers highlighted in red and bold are peak markers of QTL identified in this study and those highlighted in red are flanking markers in two LOD drop of interval; the markers in bold and italics were reported in durum wheat; the markers underlined were reported in bread wheat.

The marker order in the genetic map generated in this study was highly collinear with the durum consensus map developed by Maccaferri et al. (2015), as indicated by the Pairwise Spearman’s rank correlations (*r *= 0.992–0.999) (**Figure S4**). QTL reported for SV in the literature and identified in this study were projected onto the durum wheat consensus genetic map by projecting either a single marker near the QTL peak position or a pair of flanking markers within the QTL interval (**Table S3** and **Figure 8**). The QTL *QGlu.spa-1A* (peak marker: *wsnp_Ex_c13186_20822127*) detected in this study was projected at position 89.5 cM on chromosome 1A of the durum consensus genetic map. It is approximately 6 cM away from SSR marker *wmc312* reported to be associated with SV in durum wheat by Conti et al. (2011). The QTL *QGlu.spa-1B.1* (peak SNP: *Kukri_c38353_67*) was projected on the short arm of chromosome 1B on the durum wheat consensus map, approximately 4.4 cM away from SSR marker *gwm550* reported by Patil et al. (2009). Likewise, QTL *QGlu.spa-1B.2* (peak SNP: *Excalibur_c50079_420*) on the long arm of chromosome 1B was 6 cM away from the QTL interval (*barc181*-*psr162*) identified by Zhang et al. (2008) and Conti et al. (2011) in durum wheat, and 0.5 cM apart from the QTL (peak SNP: *CAP8_c818_370*) identified by Jernigan et al. (2018) in bread wheat. Of the two QTL reported by Roselló et al. (2018), QTL associated with marker *wPt-1140* was located within *QGlu.spa-2B.2* (peak SNP: *Kukri_c25868_56*) and another QTL associated with marker *wPt-6894* within *QGlu.spa-2B.3* (peak SNP: *Excalibur_c91034_141*). There have been no reports for QTL on the short arm of chromosome 2B close to *QGlu.spa-2B.1* (peak SNP: *RAC875_c38003_164*). A QTL on the short arm of chromosome 3A reported by Roselló et al. (2018) is about 32 cM away from *QGlu.spa-3A.1* (peak SNP: *RAC875_c64107_404*) identified in this study, indicating that these two QTL might be different and *QGlu.spa-3A.1* was a novel QTL. In addition, another QTL *QGlu.spa-3A.2* (peak SNP: *Excalibur_c14216_692* and *wsnp_Ex_rep_c69864_68824236*) on chromosome 3A was likely a novel QTL for SV.

### Identification of Putative Candidate Genes for Major Quantitative Trait Loci

**Figure 9:**
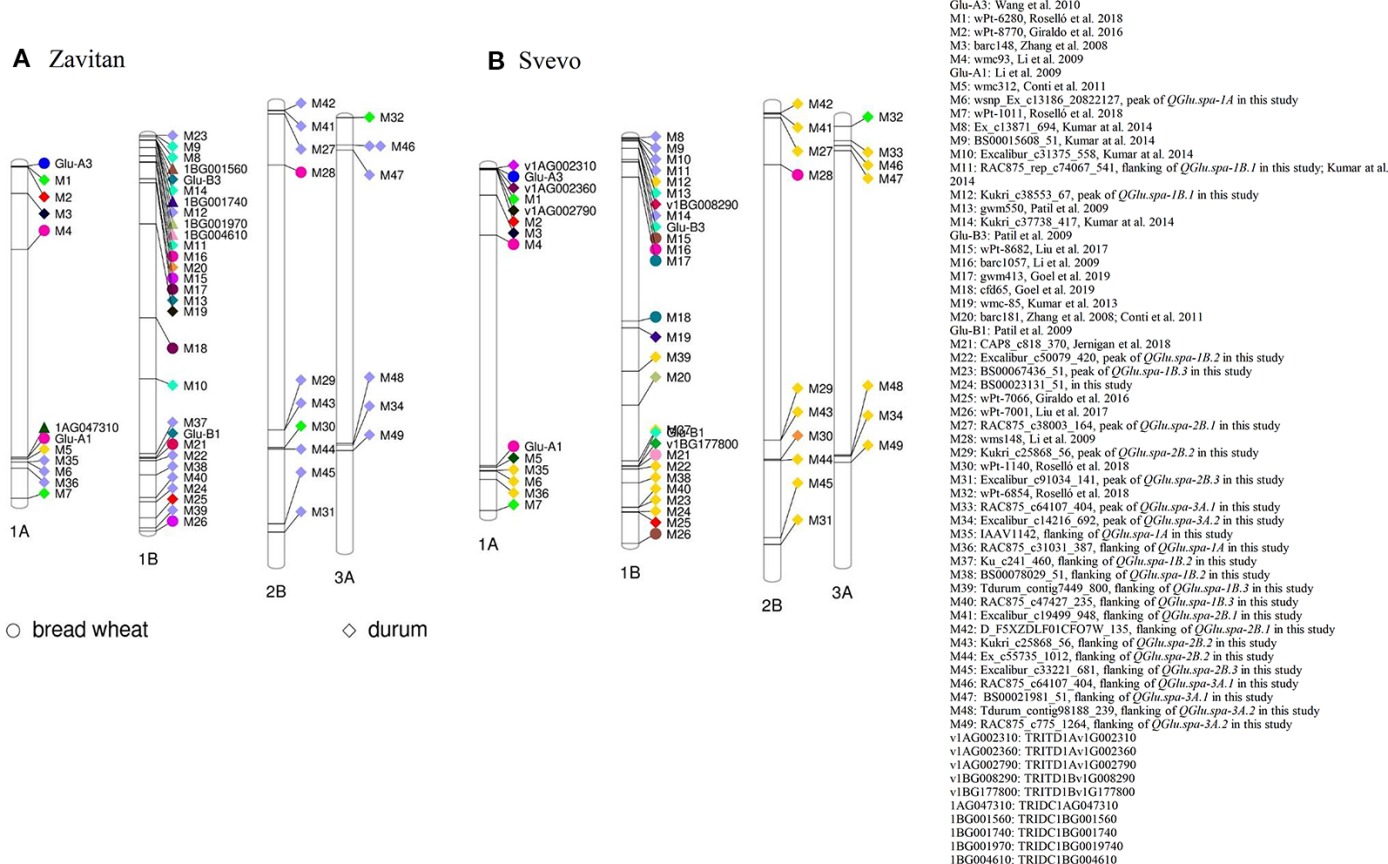
Projection of quantitative trait loci (QTL) for SDS-sedimentation volume (SV) reported in the literature (in both bread wheat and durum wheat) and the QTL identified in this study onto the reference genomes of **(A)** wild emmer wheat accession Zavitan and **(B)** durum wheat cv. Svevo.

To predict the putative candidate genes at major QTL on chromosome 1A and 1B and to facilitate comparative mapping analysis, the sequences of the peak and flanking markers associated with QTL for SV were anchored to their physical location on the genome by aligning the marker sequence to the wild emmer wheat accession Zavitan (*Triticum dicoccoides*, WEWSeq_v.1.0; Avni et al., 2017) (**Table S4** and **Figure 9A**) and the durum wheat cv. Svevo assemblies (Maccaferri et al., 2019) (**Table S4** and **Figure 9B**). The gene content in the two LOD drop of QTL region, corresponding to a region of 10.75 Mb on 1A and 11.78 Mb on 1B, was searched.

Among the annotated high confidence genes, the gene *TRIDC1AG047310* in proximity to SNP *wsnp_Ex_c13186_20822127* (M6) on chromosome 1A of Zavitan, has five transcript splice variants. Three out of five splice variants encode a protein with three domains of HMW glutenin (**Figure S5**). *TRIDC1AG047310.3* encodes a protein with sequence similarity with *Glu-A1* (GenBank accession: ANJ03342) from wild emmer wheat accession TD-256 (*T. dicoccoides*) and *Glu-1Ax1* (GenBank accession: CAA43331) from bread wheat (**Figure S6**). The HMW glutenin locus *Glu-A1* reported by Li et al. (2009) was projected on chromosome 1A where *TRIDC1AG047310* is located (**Figure 9A**). Similarly, SNP *wsnp_Ex_c13186_20822127* (M6) is about 8.66 Mb away from *Glul-A1* on chromosome 1A of durum wheat cv. Svevo (**Figure 9B**). This suggested that *TRIDC1AG047310* might be a candidate gene underlying QTL *QGlu.spa-1A*. In addition, three annotated high confidence genes (*TRITD1Av1G002310*, *TRITD1Av1G002360* and *TRITD1Av1G002790*) encode LMW-GS on the short arm of chromosome 1A of Svevo. However, no QTL was detected in this region in the present study.

The gene *TRIDC1BG001970* on chromosome 1B of Zavitan, with a distance of 108 Kb from SNP *Kukri_c38553_67* (M12), has three domains of gliadin/LMW glutenin. Three transcript splice variants were identified for gene T*RIDC1BG001970* with *TRIDC1BG001970.2* encoding a protein of 298 aa and *TRIDC1BG001970.3* for a protein of 182 aa. The transcript *TRIDC1BG001970.1* encodes a protein with 139 aa without gliadin/LMW glutenin domain (**Figure S7**). Three paralogous genes of *TRIDC1BG001970* on chromosome 1B of Zavitan were identified as *TRIDC1BG001560* (1B: 7,719,618 -7,720,229 bp), *TRIDC1BG001740* (1B: 8,606,652–8,608,041 bp) and *TRIDC1BG004610* (1B: 20,578,493–20,579,630 bp) (**Figure 9A**). However, the protein structure of *TRIDC1BG001970* is more similar to that of *TRIDC1BG001740* (**Figure S8**). *TRIDC1BG001970.2* shares 85% identity at the protein level with *Glu-B3* (GenBank # AVI69508.1) in durum cv. Langdon (**Figure S9**). On chromosome 1B of Svevo, gene *TRITD1Bv1G008290* (**Figure S9**) encoding a portion of LMW-GS is about 105 Kb away from the *Glu-B3* marker and 13.1 Mb from SNP *Kukri_c38553_67* (M12, *QGlu.spa-1B.1*). Similarly, gene *TRITD1Bv1G177800* encoding a HMW-GS is 186 Kb away from the *Glu-B1* marker and 5.88 Mb from SNP *Excalibur_c50079_420* (M22, *QGlu.spa-1B.2*) on the long arm of chromosome 1B of Svevo. This comparative mapping indicated that *TRITD1Bv1G008290* and *TRITD1Bv1G177800* are likely the candidate genes for *QGlu.spa-1B.1* and *QGlu.spa-1B.2* respectively, although the possible existence of other paralogs in these regions cannot be excluded.

## Discussion

Gluten strength is one of the most important quality criteria in durum breeding. Previous studies have shown that gluten strength of durum wheat is quantitatively controlled by a few major QTL and some minor QTL whose expression is affected by environmental conditions. In this study, a total of nine QTL were detected for gluten strength measured by SV. Two major QTL positioned on chromosome 1A and 1B were detected across all environments. These two QTL together accounted for up to 59% of the phenotypic variance. The present work also allowed the identification of a few minor QTL on chromosome 1B, 2B, and 3A, with inconsistent expression over different environments. Favorable alleles were identified from both parents at different loci.

### Quantitative Trait Loci Associated With Low Molecular Weight Glutenin Subunit

The major QTL *QGlu.spa-1B.1* explaining up to 40.1% of the phenotypic variance in the present study was identified on the short arm of chromosome 1B close to the LMW-GS locus *Glu-B3*. This finding supports the possibility that allelic variation for LMW-GS encoded by the *Glu-B3* locus on chromosome 1BS is the major contributor for the difference of gluten strength in durum wheat (Pogna et al., 1990). A major QTL on 1BS near the *Glu-B3* locus has been previously reported in a variety of durum wheat germplasm (Blanco et al., 1998; Elouafi et al., 2000; Patil et al., 2009; Kumar et al., 2013; Kumar et al., 2014), which is indicative of the importance of the *Glu-B3* region for gluten strength of durum wheat although with various levels of expression in different genetic backgrounds and environments. Apart from a strong positive correlation between the LMW-GS *Glu-B3* locus and gluten strength, a strong association of LMW-GS with pasta-cooking quality has been well documented (Pogna et al., 1990; Kovacs et al., 1995b; Ruiz and Carrillo, 1995). Similarly, nine protein alleles of the *Glu-B3* (a, b, c, d, e, f, g, h, i) with various effect on dough quality have been reported in bread wheat (Metakovsky, 1990; Gupta et al., 1991).

Strongfield and Pelissier display different profiles of LMW-GS and HMW-GS (**Figure S3**). The analysis of allele-specific PCR markers showed that there is no polymorphism for gliadin alleles *GliB1.1* and *GliB1.2* between two parents, while polymorphism exists for LMW-glutenin alleles *gluB3c* and *gluB3i* (**Table S5**). The two characteristic subunits (39,623 and 42,930 Da in Strongfield; 39,627 and 42,906 Da in Pelissier) of Glu-B3c (Wang et al., 2015) showed differential ratio in the two parents (**Figure S3**), which might result from the polymorphism of *gluB3c*. Combining with the variation of LMW-GS composition between parents, this indicated that *Glu-B3* might be associated with *QGlu.spa-1B.1* in this population. Furthermore, candidate gene analysis suggested that both *TRIDC1BG001970* and *TRIDC1BG001740* could be associated with QTL *QGlu.spa-1B.1*. The physical location of *TRIDC1BG001970* and *TRIDC1BG001740* is next to a major QTL (SNP: *Kukri_c37738_417*) contributing up to 90% of the phenotypic variation for gluten strength measured using SV in durum (Kumar et al., 2013; Kumar et al., 2014), followed by the *Glu-B3* locus on the physical sequence map of wild emmer accession Zavitan. High similarity of protein structure between the identified genes and *Glu-B3* suggested there could be a gene cluster in the *Glu-B3* region responsible for gluten strength, which is in agreement with the report that LMW-GS are encoded by multi-gene families at the *Glu-A3* and *Glu-B3* loci (D’Ovidio and Masci, 2004). Further studies are needed to clone and differentiate the functions of identified genes. In addition, a new LMW-GS allele of 43,351 Da was identified in Strongfield but not in Pelissier. Further studies are required to characterize this new allele.

### Quantitative Trait Loci Associated With High Molecular Weight Glutenin Subunit

In the present study, a major QTL (*QGlu.spa-1A*) in proximity to the HMW-GS locus *Glu-A1* on chromosome 1A was detected across all environments and explained 13.7–18.3% of variation in gluten strength. *Glu-A1* allele 1Ay was present in Strongfield but not in Pelissier (**Table S5**). The gene encoding 1Ay subunit is always silent in hexaploid wheat, while expressed in some diploid and tetraploid wheats (Jiang et al., 2009). However, no extra peak corresponding to 1Ay was detected in Strongfield by MALDI-TOF-MS, which might be due to the inactivation of the 1Ay allele. Previous studies identified similar inactivation of 1Ay allele in tetraploid wheat (Jiang et al., 2009). The variation of HMW-GS subunits 1Ax2* (**Figure S3**) is likely associated with *QGlu.spa-1A*, although no polymorphism was detected for allele 1Ax2* using PCR based analysis (**Table S5**). The discrepancy was most likely caused by the differential expression of the gene. In addition, the putative candidate gene for *QGlu.spa-1A* encodes HMW glutenin with high protein similarity to *Glu-A1*, suggesting that the genes associated with *QGlu.spa-1A* and *Glu-A1* could be the same. However, the cloning of the putative candidate gene and conversion into KASP markers are required to confirm this assumption. Likewise, a QTL for gluten strength in durum wheat was detected on chromosome 1AL, but only in one environment in a recombinant inbred line (RIL) population derived from line UC1113 and cv. Kofa (Conti et al., 2011). Another QTL associated with the HMW-GS loci detected in this study is *QGlu.spa-1B.2* on the long arm of chromosome 1B in a position close to *Glu-B1*. This result agrees with the findings reported by Conti et al. (2011) that a stable QTL associated with SV was identified on chromosome 1BL (*Glu-B1*) across multiple environments. Similarly, a QTL linked to the *Glu-B1* locus was also found to be associated with gluten strength in durum wheat (Patil et al., 2009). The subunit 1Bx7 (82,441 Da) detected only in Pelissier (**Figure S3**) is likely associated with *QGlu.spa-1B.2*. All these results confirmed the significant positive association between HMW-GS loci and gluten strength in durum wheat. QTL for SV associated with *Glu-A1* (Li et al., 2009) and *Glu-B1* (Jernigan et al., 2018) were also reported in bread wheat. However, a weaker association was reported between HMW-GS loci and gluten strength in modern durum wheat cultivars likely as a result of limited genetic variation at *Glu-1* (Sissons, 2008). A weak but significant relationship between the HMW-GS and spaghetti quality was previously reported, while some studies showed no clear relationship between these two (reviewed by Liu et al., 1996). A direct measurement of rheological properties of the dough might be needed to determine the gluten strength associated with allelic variation at *Glu-A1* and *Glu-B1* in durum wheat.

The *Glu-A1* locus presented less polymorphism compared to *Glu-B1* in both durum landraces and modern cultivars. In addition, the HMW-GS genes on chromosome lA were reported to have a negligible relationship with durum quality parameters when compared to the genes on chromosome 1B, although active *Glu-A1* alleles were found to have a favorable influence on baking properties of some Italian durum [(Liu et al., 1996) and references therein]. Conti et al. (2011) identified that the most important and stable QTL for gluten strength is associated with *Glu-B1* on chromosome 1BL. In contrast, in the present study, QTL associated with *Glu-A1* had stronger effect than the QTL at *Glu-B1* as evidenced by the higher percentage of phenotypic variance explained. The difference of the findings could be related to the genetic background of the parental lines used for the population development. A null allele at the *Glu-A1* locus was found in Mediterranean durum wheat cultivars while non-null alleles exist in about 40% of the landraces studied (Nazco et al., 2013). Likewise, over 83% of a collection of 502 durum wheat varieties from 23 countries were found to have the *GluA1c* (null) allele (Branlard et al., 1989). The presence of some alleles at the *Glu-B3* locus can offset the effect of the *Glu-B1* alleles. Removal of the *Glu-B3* effect resulted in the detection of the greatest influence of *Glu-B1* (Martínez et al., 2005). In our study, the largest effect QTL (*QGlu.spa-1B.1*) on 1BS in the *Glu-B3* region might mask the effect of *Glu-B1* alleles in some environments, although no significant interaction was observed between these two loci. Further studies are necessary to confirm the assumption and elucidate the underlying mechanism.

### Stability of Quantitative Trait Loci

High broad sense heritability of 0.96 was observed for SV in this study (**Table S2**), indicating the phenotypic variation was attributable mainly to the genetic variation. Similar high heritability value of gluten strength measured by SV has been reported in other studies carried out in durum wheat (Clarke et al., 2010; Conti et al., 2011; Kumar et al., 2013). Two stable QTL located on chromosome 1A and 1B near *Glu-A1* and *Glu-B3,* respectively, were detected across all environments tested, with the trait increasing alleles derived from Strongfield. These two QTL are highly desirable for MAS as the selected favorable alleles confer high SV in all years tested and therefore are easy to be incorporated in breeding programs. QTL × E interaction is an important contributor to the variation in the expression of complex traits. Although the genotype was the main source of variation for SV, significant QTL × E were detected from the multi-trait CIM analysis of two major QTL, *QGlu.spa-1A* and *QGlu.spa-1B.1*, which were significant across all environments and displaying fluctuations in the magnitude of the effects. Another seven QTL detected on chromosome 1B, 2B, and 3A in one or two environments had favorable alleles from Pelissier. This indicates that the expression of the alleles from Pelissier is more prone to be affected by the environment and may be favored in one environment but neutral in others. As demonstrated by previous studies, gluten strength was influenced by genotype and environment, and to some extent by the interaction of genotype × environment, suggesting trials in multiple environments are required for the selection of this trait (Patil et al., 2009; Conti et al., 2011).

Furthermore, gluten strength measured by SV could be positively correlated with GPC, which depends on the genotypes and environments (Clarke et al., 2010). The moderate positive correlation between SV and GPC and weak to moderate negative correlation between SV and GY were observed in three out of six environments in this population (**Figure S2**). However, our studies showed that the stable QTL on 1A and 1B identified in this population do not contribute to GPC and GY (data unpublished).

### Epistatic Quantitative Trait Loci Interaction

The identification of epistatic interactions for the QTL whose effects mostly dependent on the genotypes of other loci, can provide a more comprehensive understanding of genetic components controlling the expression of complex traits and a more accurate prediction for the phenotypic traits (Bocianowski, 2013). Of note, in the present study the epistatic interaction between *QGlu.spa-1A* and *QGlu.spa-1B.1* was repeatedly detected in 4 out of 6 environments indicating a positive interaction between alleles of HMW-GS and LMW-GS. Therefore, it is important to take into account such epistatic effects for marker assisted selection (MAS). Significant interactions between *Glu-B3* and other glutenin loci were observed in a previous study (Martínez et al., 2005). Likewise, QTL for gluten strength on 1BL was reported to have an epistatic effect with other loci having no main effect (Conti et al., 2011).

Taking together, *QGlu.spa-1A* and *QGlu.spa-1B.1* contributed the most desirable alleles derived from parental line Strongfield and were consistently expressed over multiple environments. Two flanking markers, Kukri c38553_67 and RCA875_rep_c74067_541, in the QTL region of *QGlu.spa-1B.1* can be used to effectively separate the DH lines into two groups with significantly different mean SV values. More distinct separation was obtained using flanking markers of both QTL *QGlu.spa-1A* (IAAV1142 and RAC875_c31031_387) and *QGlu.spa-1B.1*. The KASP assays for *QGlu.spa-1A* and *QGlu.spa-1B.1* showed the good clusters and reliable results, demonstrating the effectiveness of using these KASP markers for selecting the lines with higher SV/gluten strength in durum wheat although the validation in a diverse panel is required. As such, these KASP markers have the potential to be applied for MAS in durum breeding programs. These two QTL should be subjected to map-based cloning. Although the candidate genes were predicted for these two QTL based on the QTL position on the reference genomes of durum wheat cv. Svevo and wild emmer accession Zavitan along with the comparison with previously published studies, further studies are needed to confirm our assumption. Haplotype analysis of these two QTL along with another two QTL on 3A indicated the DH lines with the combination of all favorable alleles at each QTL had the highest mean SV across all environments. These DH lines are the good candidates as parental lines for developing new varieties with strong gluten strength. Similar haplotype analysis of QTL*QGlu.spa-1A* and *QGlu.spa-1B.1* in a diverse durum panel will enhance our understanding of the allelic variants of *Glu-A1* and *Glu-B3* and may facilitate more effective use of favorable alleles in further improving gluten strength of durum wheat.

## Data Availability Statement

All datasets generated for this study are included in the article/**Supplementary Material**.

## Author Contributions

YR and RK conceptualized this study. YR, RK, AS, RD and RC generated the population and implemented the field trials and phenotyping of the population. IP and PG conducted the marker validation and the KASP assays. WZ, AS and PF provided the genotyping platform. BY conducted data analysis. BY and YR contributed to data management and visualization. BY wrote the original manuscript and YR, RK, RD, and PF contributed to the review and editing of the manuscript. YR was the principal investigator and acquired the fund for this study.

## Funding

Financial support was received from the National Wheat Improvement Program and the Canadian Agricultural Partnership with support from Agriculture and Agri-Food Canada, Western Grains Research Foundation, Alberta Wheat Commission, Saskatchewan Wheat Development Commission, and Manitoba Wheat and Barley Growers Association. The work was also supported by Canadian Wheat Improvement Flagship funded by National Research Council Canada.

## Conflict of Interest

The authors declare that the research was conducted in the absence of any commercial or financial relationships that could be construed as a potential conflict of interest.
