# Supplementary material for: High Density Mapping of Quantitative Trait Loci Conferring Gluten Strength in Canadian Durum Wheat
Source: Front Plant Sci. 2020 Mar 4;11:170. doi: 10.3389/fpls.2020.00170 (PMC7064722; doi:10.3389/fpls.2020.00170)
Supplement: Supplementary file 1 [file DataSheet_1.pdf]

High Density Mapping of Quantitative Trait Loci Conferring Gluten Strength in Canadian Durum Wheat

Yuefeng Ruan[1], Bianyun Yu[2]*, Ron E. Knox[1], Asheesh K. Singh[1†], Ron DePauw1[†], Richard Cuthbert[1], Wentao Zhang[2], Isabelle Piche[1], Peng Gao[2], Andrew Sharpe[2†] and Pierre Fobert[3]

[1]Swift Current Research and Development Centre, Agriculture and Agri-Food Canada, Swift Current, SK, Canada,
[2] Aquatic and Crop Resource Development, National Research Council Canada, Saskatoon, SK, Canada,
[3] Aquatic and Crop Resource Development, National Research Council Canada, Ottawa, ON, Canada

***Correspondence:**

Bianyun Yu

Bianyun.Yu@nrc-cnrc.gc.ca

Key words: durum wheat, gluten strength, SDS-sedimentation volume, sodium dodecyl sulphate-sedimentation volume, quantitative trait loci, single nucleotide polymorphism

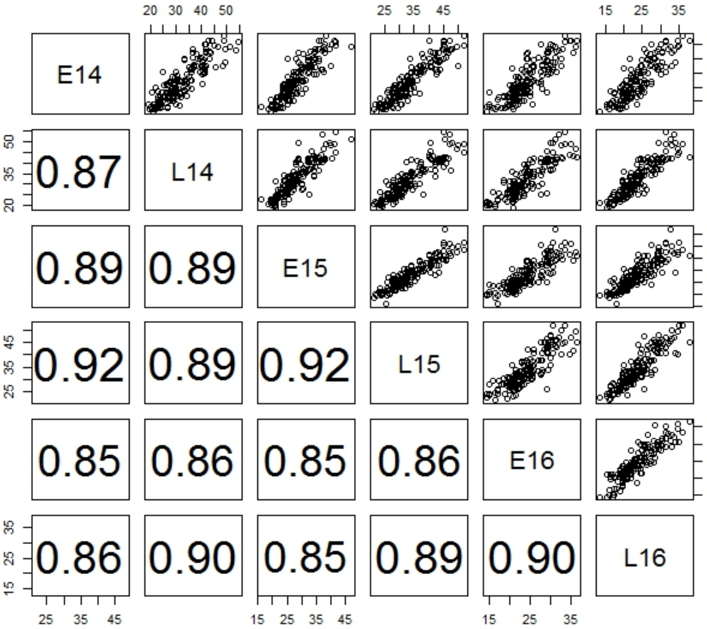

Supplementary Figure S1 Pearson's correlation of SDS-sedimentation volume (SV) in DH lines of Pelissier × Strongfield across environments at significance level $p < 0.001$. E, early seeding date; L, late seeding date.

A

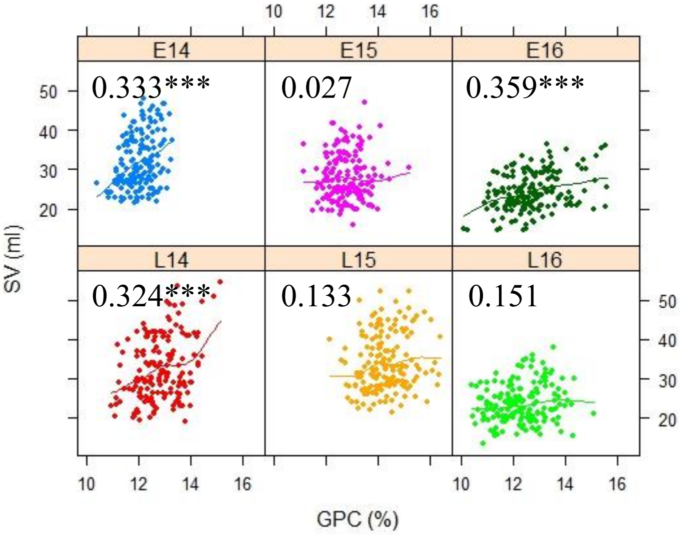

B

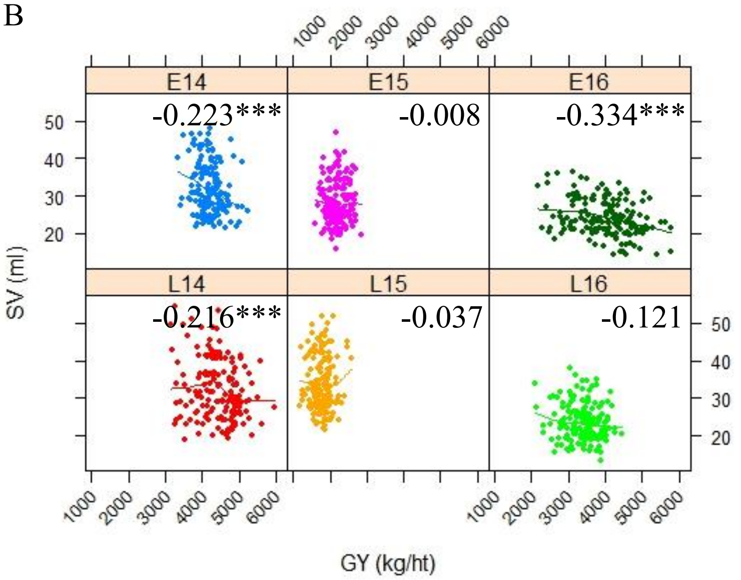

Supplementary Figure S2 Pearson's correlation of SDS-sedimentation volume (SV) and grain protein concentration (GPC) (A), SV and grain yield (GY) (B) in DH lines of Pelissier × Strongfield across environments. E, early seeding date; L, late seeding date. ***, significance level $p < 0.001$.

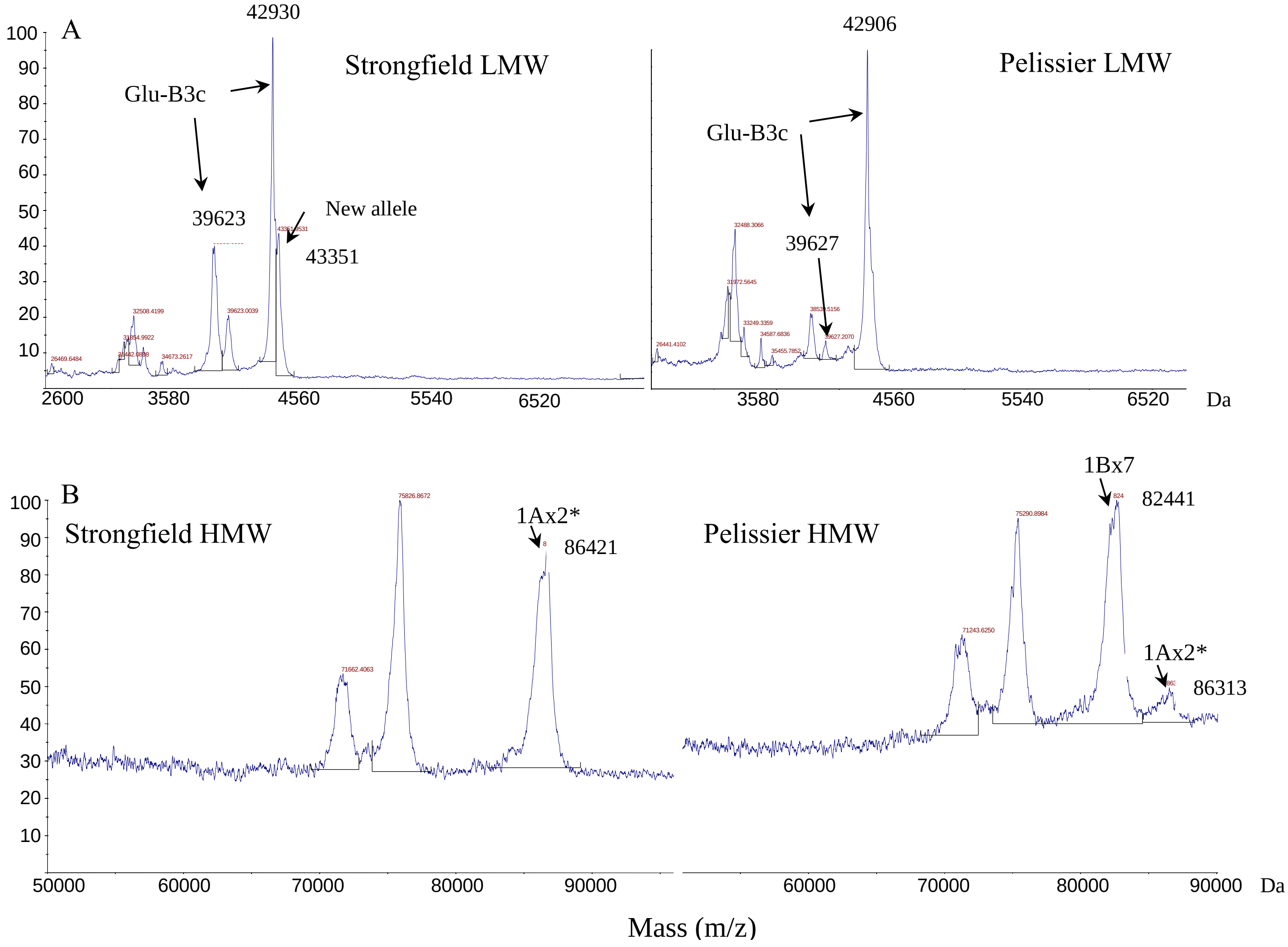

Supplementary Figure S3 MALDI-TOF-MS spectrum patterns of (A) LMW-GS and (B) HMW-GS of Strongfield and Pelissier.

Reference

Glu-B3c: Wang et al. 2015. PLoS ONE 10(9): e0138981. https://doi.org/10.1371/journal.pone.0138981

1Ax2*: Liu et al. 2009. J cereal Sci 50:295-301

1Bx7: Gao et al. 2010. J Agric Food Chem 58:2777-2786

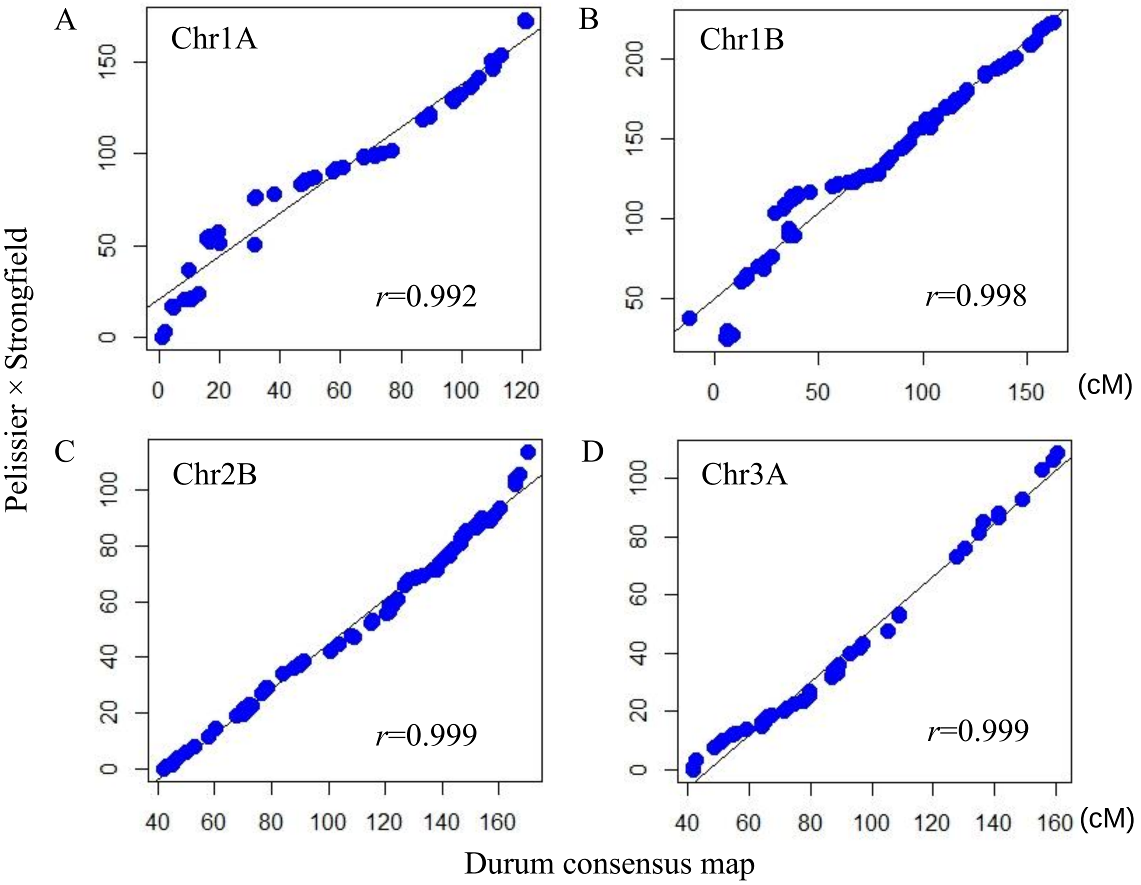

Supplementary Figure S4 Scatterplot displaying the position of common markers on chromosome (A) 1A, (B) 1B, (C) 2B and (D) 3A of the durum consensus map and the genetic map generated in this study. Each dot represents a marker that is common between the two maps. *r*, Spearman rank correlation at $p < 2.2e\text{-}16$.

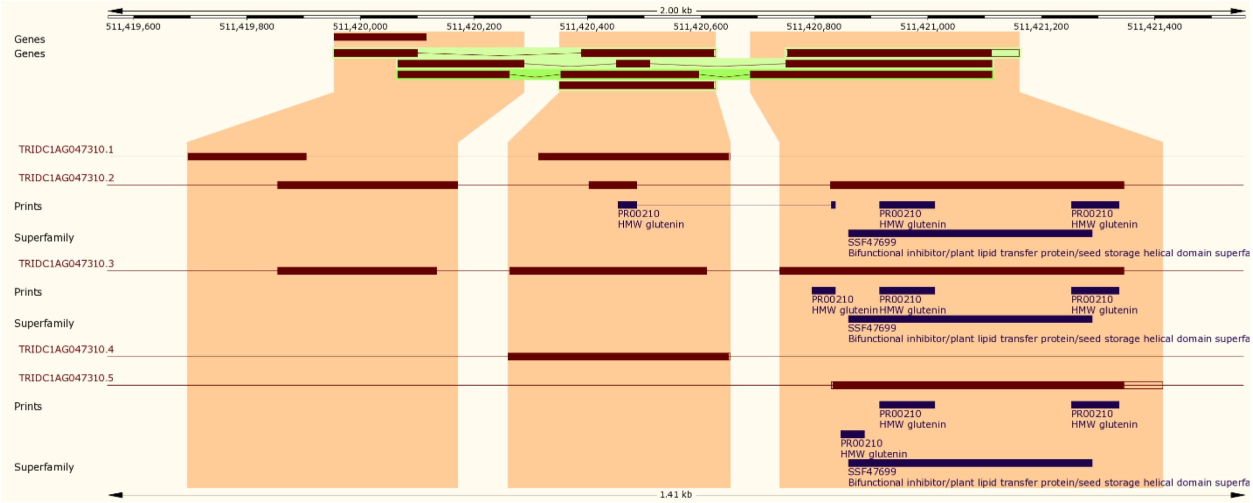

Supplementary Figure S5 Transcript splice variants of gene *TRIDC1AG047310* in wild emmer wheat accession Zavitan. The filled red blocks represent the coding regions; the lines between the red filled bars are introns; the filled blue blocks represent the conserved domains. Prints, protein fingerprints (groups of conserved motifs) from the PRINTS database. Superfamily, protein domains and motifs from the SUPERFAMILY database.

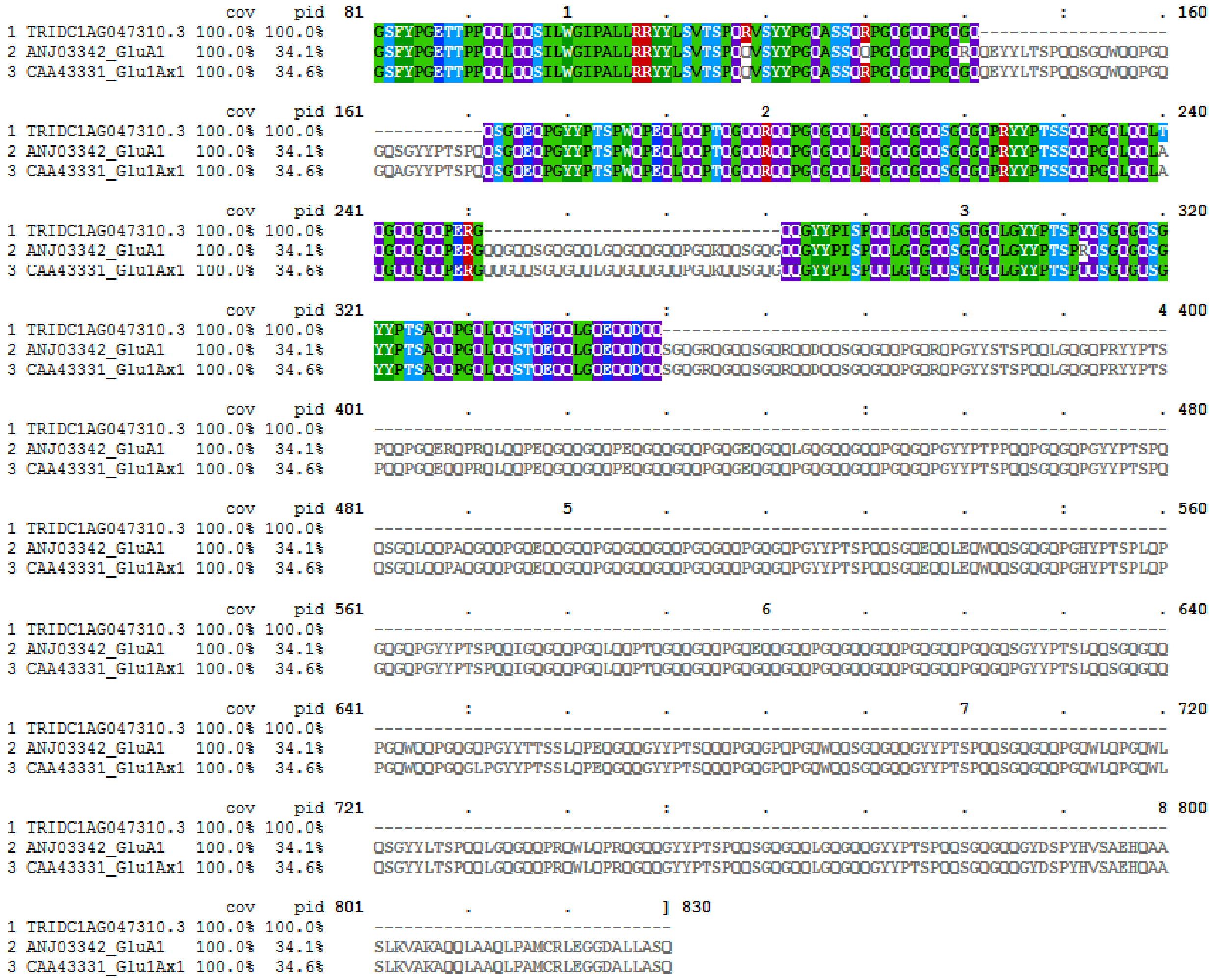

Supplementary Figure S6 Multiple alignment of protein sequences of *TRIDC1AG047310.3* from *Triticum dicoccoides* wild emmer wheat accession Zavitan with *Glu-A1* (GenBank# ANJ03342) from *Triticum dicoccoides* wild emmer wheat accession TD-256 and *Glu1Ax1* (GenBank# CAA43331) from bread wheat cultivar 'Hope'. Sequence alignment was conducted using Clustal Omega, https://www.ebi.ac.uk/Tools/msa/clustalo/.

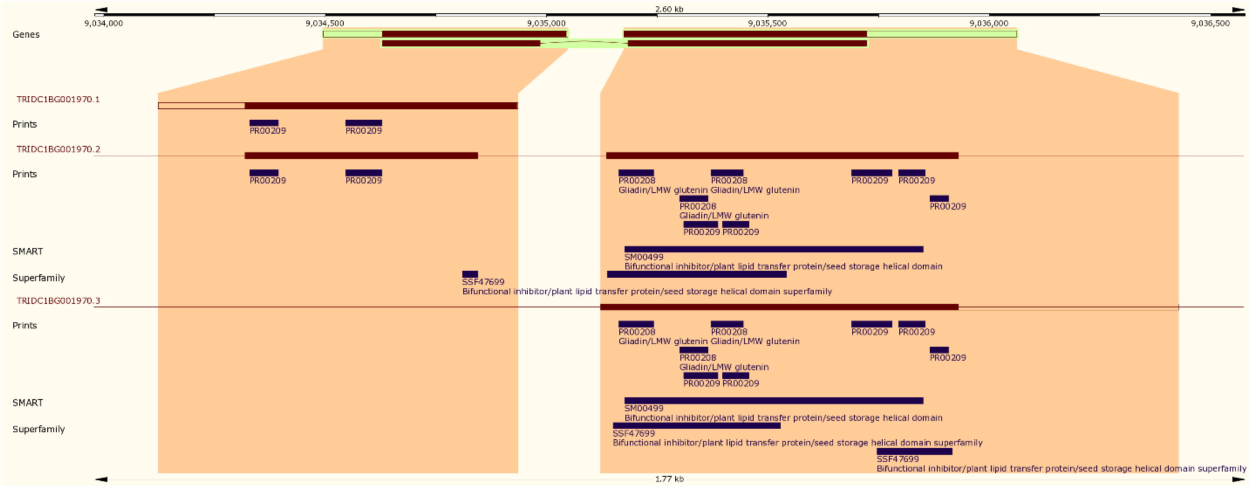

Supplementary Figure S7 Transcript splice variants of gene *TRIDC1BG001970* in wild emmer wheat accession Zavitan. The filled red blocks represent the coding regions; the lines between the red filled bars are introns; the unfilled red blocks represent the untranslated regions (UTR); the filled blue blocks represent the conserved domains. Prints, protein fingerprints (groups of conserved motifs) from the PRINTS database. Superfamily, protein domains and motifs from the SUPERFAMILY database.

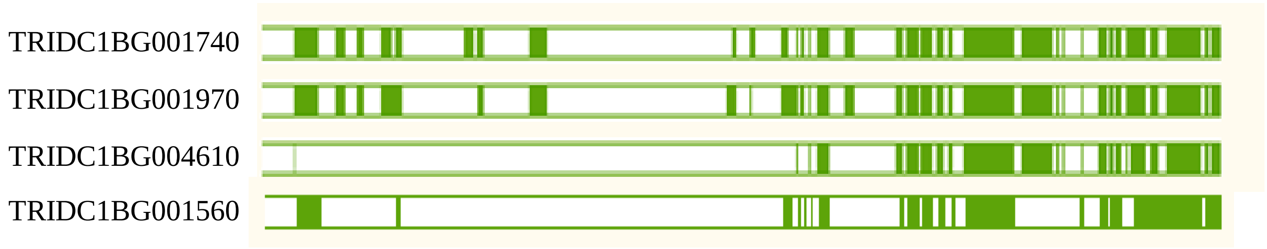

Supplementary Figure S8 Protein structure of *TRIDC1BG001970* and three paralogs in wild emmer wheat accession Zavitan.

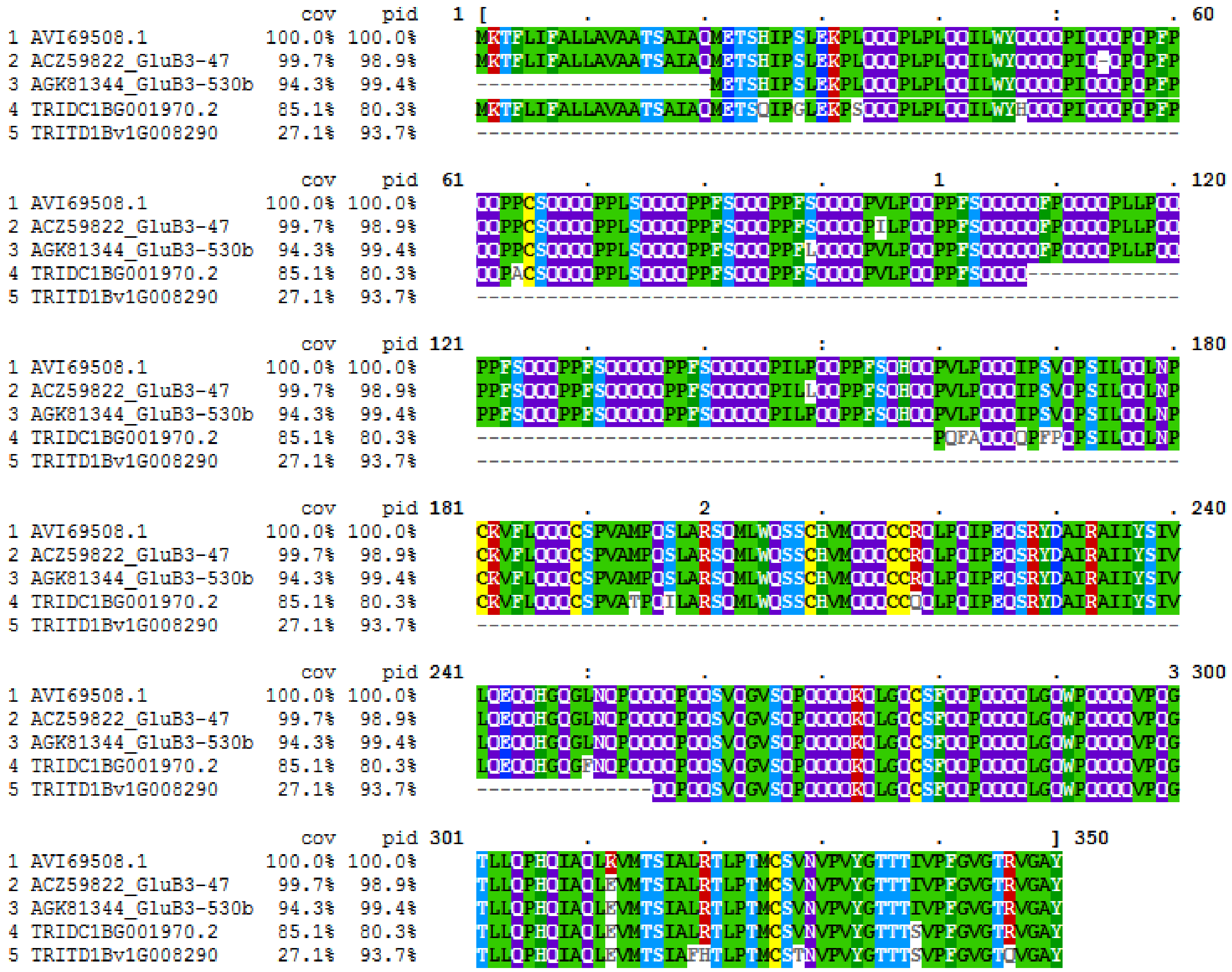

Supplementary Figure S9 Multiple alignment of protein sequences of *TRIDC1BG001970.2* from wild emmer wheat accession Zavitan with *Glu-B3* from Triticum turgidum ssp. durum cv. Langdon (GenBank # AVI69508.1), Triticum dicoccon (GenBank # ACZ59822), bread wheat (GenBank # AGK81344), and durum wheat cv. Svevo (*TRITD1Bv1G008290*). Sequence alignment was conducted using Clustal Omega, https://www.ebi.ac.uk/Tools/msa/clustalo/.

Supplementary Table S1 Means of parents and population, and range, coefficient of variation (CV) and repeatability of SDS-sedimentation volume (SV) for the population.

		DH lines				Parents		
**Year**	**Env**	**Mean±SD**	**Range (Min-Max)**	**CV (%)**	**R**	**Strongfield**	**Pelissier**	**Dif**
2014	E14	32.0±6.8	21.5-58.0	0.21	0.93	41.5	32.8	****
	L14	32.6±8.0	19.0-54.5	0.25		37.8	30.5	**
2015	E15	28.1±5.9	16.0-47.0	0.21	0.95	30.5	26.8	ns
	L15	34.0±7.1	21.5-52.0	0.21		37.8	30.0	***
2016	E16	24.3±4.8	14.5-36.5	0.20	0.95	24.2	25.3	ns
	L16	23.7±4.9	13.5-38.0	0.21		27.2	23.0	**

Env, Environment; SD, standard deviation; Min, Minimum; Max, Maximum; CV, coefficient of variation; R, Repeatability; Dif, Difference
*,**,***,**** $p<0.05$, $p<0.01$, $p<0.001$, $p<0.0001$, respectively; ns: not significant.

Supplementary Table S2 Analysis of variance (ANOVA) of SDS-sedimentation volume (SV) and its heritability estimate.

Effect	Variance	Heritability
Line	34.01****	0.96
Year	16.85	
Line*Year	1.66****	
Seeding date	0	
Seeding date (Year)	5.71	
Line*Seeding date	0.33	
Line*Year*Seeding date	1.31***	
Replication (Year Seeding date)	0.57*	
Residual	6.10****	

*,**,***,**** $p<0.05$, $p<0.01$, $p<0.001$, $p<0.0001$, respectively.

Supplementary Table S3 Position of each QTL associated marker on the durum consensus map developed by Maccaferri et al. (2015).

Chr	Marker	Position (cM)	QTL Source
1A	wPt-8770	2.6	Giraldo et al. 2016
1A	wPt-6280	5.7	Roselló et al. 2018
1A	wmc95	22.3	Conti et al. 2011
1A	barc148	39.9	Zhang et al. 2008
1A	BM140362	66.9	Zhang et al. 2008
1A	BM140362_603	66.9	Conti et al. 2011
1A	wmc312	83.7	Conti et al. 2011
1A	wsnp_Ex_c13186_20822127	89.5	in this study
1A	IAAV1142	89.5	in this study
1A	RAC875_c31031_387	97.3	in this study
1A	wPt-6754	142.1	Roselló et al. 2018
1A	wPt-1310	167.4	Roselló et al. 2018
1A	wPt-6853	167.4	Roselló et al. 2018
1A	wPt-1011	167.4	Roselló et al. 2018
1B	Kukri_c38553_67	-12.4	in this study
1B	gwm550	-8	Patil et al. 2009
1B	wmc85	8.3	Kumar et al. 2013
1B	wPt-8682	25.1	Liu et al. 2017
1B	wPt-5562	28.9	Liu et al. 2017
1B	gwm413	29.9	Goel et al. 2018
1B	cfd65	36.7	Goel et al. 2018
1B	barc181	53.4	Zhang et al. 2008
1B	barc181	53.4	Conti et al. 2011
1B	psr162	76.7	Zhang et al. 2008
1B	psr162	76.7	Conti et al. 2011
1B	Ku_c241_460	78.6	in this study
1B	CAP8_c818_370	82.2	Jernigan et al. 2018
1B	Excalibur_c50079_420	82.7	in this study
1B	cfa2129	83.6	Conti et al. 2011
1B	BS00078029_51	90.7	in this study
1B	BS00067436_51	109	in this study
1B	RAC875_c47427_235	109	in this study
1B	wPt-7066	116.7	Giraldo et al. 2016
2B	RAC875_c38003_164	17.7	in this study
2B	RAC875_c28185_91	115	in this study
2B	wPt-1140	121.9	Roselló et al. 2018
2B	Ex_c55735_1012	122.2	in this study
2B	Ku_c12037_482	123.4	in this study
2B	wPt-6894	163.8	Roselló et al. 2018
3A	wPt-6854	9.6	Roselló et al. 2018
3A	RAC875_c64107_404	41.6	in this study
3A	BS00021981_51	48.4	in this study
3A	Tdurum_contig98188_239	88.7	in this study
3A	Excalibur_c14216_692	89.1	in this study

Supplementary Table S4 Position of each QTL associated marker on the reference genome of wild emmer wheat Zavitan and durum cv. Svevo.

Marker	Chr	Reference	Species	Zavitan		Svevo		Annotation
				Start (bp)	End (bp)	Start (bp)	End (bp)	
Glu-A3	1A	Wang et al. 2010	bread wheat	4387840	4388091	5047538	5047789	Glu-A3
wPt-6280	1A	Rosello et al. 2018	durum	5852494	5852890	5810671	5811067	M1
wPt-8770	1A	Giraldo et al. 2016	durum	6536132	6537156	7126288	7127312	M2
barc148	1A	Zhang et al. 2008	durum	52860532	52860515	50183000	50183539	M3
wmc93	1A	Li et al. 2009	bread wheat	150121066	150121210	116504885	116505029	M4
Glu-A1	1A	Li et al. 2009	bread wheat	511420468	511419792	500862023	500861444	Glu-A1
wmc312	1A	Conti et al. 2011	durum	514223301	514223319	503575801	503575933	M5
wsnp_Ex_c13186_20822127	1A	in this study	durum	520493503	520493689	509524198	509524384	M6
wPt-1011	1A	Rosello et al. 2018	durum	583275392	583274855	575534355	575533818	M7
Ex_c13871_694	1B	Kumar at al. 2014	durum	1654670	1654770	1901533	1901445	M8
BS00015608_51	1B	Kumar at al. 2014	durum	415393	415467	3290899	3290806	M9
Excalibur_c31375_558	1B	Kumar at al. 2014	durum	423247404	423247370	4737575	4737675	M10
RAC875_rep_c74067_541	1B	in this study; Kumar at al. 2014	durum	20705721	20705821	6309319	6309419	M11
Kukri_c38553_67	1B	in this study	durum	8926221	8926121	6366035	6366135	M12
gwm550	1B	Patil et al. 2009	durum	81889311	81889326	7137590	7137609	M13
Kukri_c37738_417	1B	Kumar at al. 2014	durum	7895459	7895559	19411152	19411192	M14
Glu-B3	1B	Patil et al. 2009	durum	7894973	7895713	19411257	19411764	Glu-B3
wPt-8682	1B	Liu et al. 2017	bread wheat	46600484	46601247	38783767	38783004	M15
barc1057	1B	Li et al. 2009	bread wheat	35194256	35194236	42757128	42757108	M16
gwm413	1B	Goel et al. 2018	bread wheat	75690863	75690843	68064894	68064874	M17
cfd65	1B	Goel et al. 2018	bread wheat	317615636	317615655	307258796	307258815	M18
wmc-85	1B	Kumar et al. 2013	durum	153772948	153772930	318500692	318500808	M19
barc181	1B	Zhang et al. 2008; Conti et al. 2011	durum	45949037	459490033	447666581	447666237	M20
Glu-B1	1B	Patil et al. 2009	durum	560805752	560804078	548285742	548284068	Glu-B1
CAP8_c818_370	1B	Jernigan et al. 2018	bread wheat	562623253	562623353	550507283	550507383	M21
Excalibur_c50079_420	1B	in this study	durum	566536605	566536705	554352851	554352948	M22
BS00067436_51	1B	in this study	durum	no hit on 1B	no hit on 1B	618405757	618405857	M23
BS00023131_51	1B	in this study	durum	637633534	637633621	625310175	625310274	M24
wPt-7066	1B	Giraldo et al. 2016	durum	666028853	666028835	626706098	626706686	M25
wPt-7001	1B	Liu et al. 2017	bread wheat	688200854	688200961	678110594	678110701	M26
RAC875_c38003_164	2B	in this study	durum	19466815	19466914	23313699	23313798	M27
wms148	2B	Li et al. 2009	bread wheat	107880470	107880451	101237709	101237690	M28
Kukri_c25868_56	2B	in this study	durum	568714900	568714994	560834228	560834322	M29
wPt-1140	2B	Rosello et al. 2018	durum	599975768	599976323	592441176	592441731	M30
Excalibur_c91034_141	2B	in this study	durum	746556483	746556583	735035921	735036021	M31
wPt-6854	3A	Rosello et al. 2018	durum	949244	949735	15590266	15589784	M32
RAC875_c64107_404	3A	in this study	durum	48327548	48327648	39304973	39305073	M33
Excalibur_c14216_692	3A	in this study	durum	570549213	570549313	565614710	565614958	M34
TRITD1Av1G002310	1A					5047553	5048723	1Av1G002310
TRITD1Av1G002360	1A					5125896	5256346	1Av1G002360
TRITD1Av1G002790	1A					6157475	6158365	1Av1G002790
TRITD1Bv1G008290	1B					19517003	19518220	1Bv1G008290
TRITD1Bv1G177800	1B					548472249	548528699	1Bv1G177800
IAAV1142	1A	in this study	durum	520380641	520380841	509420713	509420913	M35

RAC875_c31031_387	1A	in this study	durum	531133923	531134011	523844292	523844380	M36
Ku_c241_460	1B	in this study	durum	553413778	553413878	540904008	540904083	M37
BS00078029_51	1B	in this study	durum	591197604	591197696	578032590	578032682	M38
Tdurum_contig7449_800	1B	in this study	durum	682850297	682850397	391204986	391208086	M39
RAC875_c47427_235	1B	in this study	durum	630021035	630021135	618264413	618264513	M40
Excalibur_c19499_948	2B	in this study	durum	13700845	13700920	16957157	16957232	M41
D_F5XZDLF01CFO7W_135	2B	in this study	durum	12467447	12467469	15052270	15052292	M42
Kukri_c25868_56	2B	in this study	durum	568714919	568714994	560834247	560864322	M43
Ex_c55735_1012	2B	in this study	durum	602435193	602435254	594972129	594972190	M44
Excalibur_c33221_681	2B	in this study	durum	732293642	732293717	723084325	723084400	M45
RAC875_c64107_404	3A	in this study	durum	48327548	48327623	47956408	47956483	M46
BS00021981_51	3A	in this study	durum	57153647	57153747	56832502	56830602	M47
Tdurum_contig98188_239	3A	in this study	durum	568417973	568418068	563478011	563478106	M48
RAC875_c775_1264	3A	in this study	durum	580552562	580552662	576042320	576042420	M49
TRIDC1AG047310	1A			511419954	511421160			1AG047310
TRIDC1BG001560	1B			7719618	7720229			1BG001560
TRIDC1BG001740	1B			8606652	8608041			1BG001740
TRIDC1BG001970	1B			9034496	9036061			1BG001970
TRIDC1BG004610	1B			20578493	20579630			1BG004610

Supplementary Table S5 Polymorphic screening of published molecular markers of Gliadin and Glutenin between parents of the population used in this study.

Trait	Marker	Target Alleles	Strongfield amplicon (bp)	Pelissier amplicon (bp)	Reference
**Gliadin**	*GliB1.1*	Glu-B3b	375[a]	375	Zhang, et al. 2003; Liu, et al. 2014
	GliB1.2	Glu-B3a, B3h	419	419	Zhang, et al. 2003; Liu, et al. 2014
**HMW Glutenin, *Glu-A1***	*GluA1*	Ax2*/Ax1	380	380	Liu, et al. 2008
	Xid3	1Ay	780	no band	Dong, et al. 2017
	Xid4	1Ax	167	167	Dong, et al. 2017
	Xrj5		625	625	Dong, et al. 2017
	Xrj6	resides between 1Ay and 1Ax genes	690	690	Dong, et al. 2017
	Xrj7	located downstream of 1Ax	140	140	Dong, et al. 2017
**HMW Glutenin, *Glu-B1***	*Bx7^{OE}*	Bx7^{OE}	514	514	Butow, et al. 2004
**LMW Glutenin, *GLu-B3***	*gluB3a*	a	no band	no band	Wang, et al. 2009
	gluB3b	b	no band	no band	Wang, et al. 2009
	gluB3c	c	800	700	Wang, et al. 2009
	gluB3d	d	380	380	Wang, et al. 2009
	gluB3e	e	no band	no band	Wang, et al. 2009
	gluB3fg	fg[b]	no band	no band	Wang, et al. 2009
	gluB3g	g	no band	no band	Wang, et al. 2009
	gluB3h	h	no band	no band	Wang, et al. 2009
	gluB3i	i	no band	> 1000 bp	Wang, et al. 2009
	gluB3bef	bef[c]	heterogeneous (no band, 400, 400/250)	heterogeneous (no band, 400, 250)	Wang, et al. 2009

[a]Amplicon size (bp) by the corresponding marker.
[b]Specific for *Glu-B3f* and *g* alleles.
[c]Specific for *Glu-B3b, e* and *f* alleles.

References:

Butow, B. J., et al. (2004). Theoretical and Applied Genetics 109: 1525-1535.
Dong, Z., et al. (2017). PLoS ONE 12(7): e0180766.
Liu, S., et al. (2008). Theoretical and Applied Genetics 118: 177.
Liu, S., et al. (2014). Crop Science 54: 1304-1321.
Wang, L. H., et al. (2009). Theoretical and Applied Genetics 118: 525-539.
Zhang, W., et al. (2003). Theoretical and Applied Genetics 107: 130-138.
